# Cellular Composition of the Brain of a Northern Minke Whale

**DOI:** 10.1002/cne.70089

**Published:** 2025-09-18

**Authors:** Kamilla Avelino‐de‐Souza, Nina Patzke, Karl Æ. Karlsson, Paul R. Manger, Suzana Herculano‐Houzel

**Affiliations:** ^1^ Instituto de Ciências Biomédicas Universidade Federal do Rio de Janeiro Rio de Janeiro Brazil; ^2^ Faculty of Medicine, Institute for Mind, Brain and Behavior HMU Health and Medical University Potsdam Germany; ^3^ Biomedical Engineering Reykjavik University Reykjavik Iceland; ^4^ School of Anatomical Sciences University of the Witwatersrand Johannesburg South Africa; ^5^ Department of Psychology Vanderbilt University Nashville Tennessee USA; ^6^ Vanderbilt Brain Institute Vanderbilt University Nashville Tennessee USA

**Keywords:** brain evolution, brain size, cerebral cortex, cetaceans, glia/neuron ratio, Mysticeti, number of neurons, RRID:AB_11204707

## Abstract

The largest mammalian brains belong to cetacean species among the cetartiodactyls. Stereological analyses have estimated cetacean numbers of cerebral cortical neurons to be more than the average 16 billion of humans, yet isotropic fractionator estimates in artiodactyls predict that even the largest cetacean brains should have no more than a few billion cortical neurons. Here, we used the isotropic fractionator to investigate these contrasting estimates of neuronal numbers by determining the numbers of neurons and non‐neuronal cells forming the brain of a northern minke whale, previously estimated using stereology as containing 12.8 billion cortical neurons (Eriksen and Pakkenberg 2007), and comparing it to our dataset of several dozen mammalian species analyzed with the same method. We report that, with 3.2 billion neurons, the minke whale cerebral cortex conforms to the quantitative scaling rules that apply to other mammals, especially the closely related artiodactyls. The same brain contained a total of 57.4 billion neurons, of which 54.2 billion were cerebellar neurons, matching the expected numbers of a hypothetical artiodactyl brain of similar cerebellar mass. In addition, we found that the northern minke whale brain, with a mass of 2683.9 g, contained 173.4 billion non‐neuronal cells, following the universal scaling rules that apply to the brain in all therian mammals examined to date. Thus, how non‐neuronal cells are added to the mammalian brain is conserved across therian mammals and is not affected by the transition to an obligatory aquatic life history. Strikingly, we find that the minke whale is an outlier amongst mammals in having almost 18 cerebellar neurons for every neuron in the cerebral cortex, compared to the average ratio of 4, which might be related to infrasonic communication. In addition, with only approximately 88 million neurons, the remainder of the brain (brainstem/diencephalon/subcortical telencephalon) of the northern minke whale exhibited the lowest relative neuronal density of these regions reported in mammalian brains, which might be related to the absence of limbs compared to all other mammalian species. Our results indicate that the number of neurons in cetacean brains has been grossly overestimated by stereological accounts, and place whale brains on par with highly cognitively capable macaws, macaques, baboons, and elephants, but below great apes and humans, in terms of numbers of cortical neurons.

## Graphical abstract

Avelino‐de‐Souza et al. show that the minke whale has 3.2 billion neurons in the cerebral cortex, as predicted for a generic cetartiodactyl species, which places it and other cetaceans between monkeys and great apes in a ranking of mammal and bird species by total numbers of neurons in the pallium/cerebral cortex.

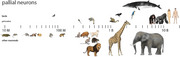

## 1 Introduction

Cetaceans (whales, dolphins, and porpoises) are obligatory aquatic mammals that diverged from their terrestrial relatives approximately 50 million years ago, in the process evolving adaptations related to survival in the marine environment (Perrin et al. 2009; Thewissen and Williams 2002; Uhen 2010). The infraorder Cetacea is composed of two parvorders: The Odontoceti (toothed whales) and Mysticeti (filter‐feeding whales) (Uhen 2010). Based on molecular (Gatesy 1997; Montgelard et al. 1997; Shimamura et al. 1997; Matthee et al. 2001) and morphological phylogenetic analyses (Gingerich et al. 2001; Thewissen et al. 2001), cetaceans have been aligned with the infraorder Artiodactyla (even‐toed ungulates) and grouped as the order Cetartiodactyla.

During the transition to an aquatic lifestyle, cetaceans underwent key transformations to their morphophysiology, with significant changes in neuromorphology only occurring 20 million years after becoming fully aquatic (Perrin et al. 2018; Manger 2006). The modern cetacean fauna (the Neocete) includes species with the largest brains (Marino 2002) and the most gyrencephalic cortices (Manger et al. 2012) observed. How and why cetaceans evolved large brains, especially as this is presumed to incur a high metabolic cost (Aiello and Wheeler 1995), making it unlikely to be a neutral shift with respect to overall fitness (Montgomery et al. 2013), is a currently unresolved question. Species within the two extant cetacean parvorders do not differ significantly in absolute brain size; however, there is a dramatic difference in their relative brain mass (in respect to body mass), with Mysticetes generally having significantly smaller relative brain sizes than Odontocetes (Montgomery et al. 2013), although the Odontocete sperm whale, with an almost 8 kg brain, has a relative brain size similar to that observed in the Mysticetes (Manger 2006).

The most often forwarded hypothesis proposes that the evolution of large brains (in both absolute and relative size) in cetaceans was driven by the need for complex cognitive abilities required for survival in an increasingly complex social environment (Marino et al. 2007, 2004, 2008; Connor 2007). Alternatively, the thermogenic hypothesis proposed that cetaceans evolved large brains independently of any cognitive demands in response to oceanic cooling during the Eocene‐Oligocene transition (Manger 2006, 2013; Manger et al. 2021), rather as a response that allowed cetacean neural processes to withstand the high rate of heat loss experienced in water (Manger 2006), which is estimated to be 90 times faster than in air at the same ambient temperature (Downhower and Blumer 1988).

It has been proposed that the cognitive advantage of large‐brained primates, including humans, compared to other mammals of similar brain size, is primarily the result of much larger numbers of cortical neurons that occur at a higher neuronal density in primate cortices in comparison to similar‐sized cortices in non‐primate mammalian orders, such as afrotherians, artiodactyls, eulipotyphlans, glires, and marsupials (Herculano‐Houzel 2016). The difference is not due to an evolutionary increase in neuronal density in primate cortices, but simply to a lack of major decreases in neuronal density as primate cortices gain neurons, in contrast to the systematic drop in neuronal density as non‐primate cortices gain neurons (Herculano‐Houzel, Catania, et al. 2015; Dos Santos et al. 2017; Jardim‐Messeder et al. 2017). It appears that the scaling of neuronal numbers within the cerebral cortex with increasing average neuronal cell volume (which translates as decreased neuronal density; Mota and Herculano‐Houzel 2014) was conserved during the evolutionary history of non‐primate mammals, whereas primates appear to have diverged with their own phylogenetically constrained scaling law (Herculano‐Houzel, Catania, et al. 2015). In contrast, all mammalian species examined so far, including primates, share the relationship between the mass of brain (or brain structures) and numbers of non‐neuronal cells, meaning that brains of similar mass have similar numbers of non‐neuronal cells, and that non‐neuronal cell densities remain approximately constant across species, regardless of brain size or number of neurons (Herculano‐Houzel and Dos Santos 2018). This putative pan‐mammalian scaling rule of the numbers of non‐neuronal cells suggests that there are phylogenetic or functional constraints that govern the addition of non‐neuronal cells in mammalian brains (Mota and Herculano‐Houzel 2014; Herculano‐Houzel, Manger, et al. 2014), while neurons are free to vary in size (Herculano‐Houzel and Dos Santos 2018).

As absolute numbers of cortical neurons correlate to cognitive capabilities in primates and birds (Herculano‐Houzel 2017; Ströckens et al. 2022), the putative evolution of large cetacean brains driven by a need for “complex” cognitive abilities (Marino 2004) implies that cetaceans have extraordinarily large numbers of cortical neurons. That expectation is, in principle, supported by claims based on stereological analyses that the cerebral cortex of the minke whale, harbor porpoise, and pilot whale consists of an astonishing 13, 15, and over 37 billion neurons, respectively (Eriksen and Pakkenberg 2007; Walloe et al. 2010; Mortensen et al. 2014). In contrast, the human cerebral cortex has, on average, 16 billion (Azevedo et al. 2009), twice as many as the second largest primate brains, the gorilla and orangutan (Herculano‐Houzel and Kaas 2011). Our previous quantitative analysis of the cellular composition of artiodactyl brains using the isotropic fractionator allowed us to predict that even the large cerebral cortex of the pilot whale, about twice as large as the human cortex, would be composed of only around 3 billion neurons (Kazu et al. 2014). If true, the stereological estimates would make cetaceans (and not humans) exceptional outliers in brain evolution and negate the ongoing hypothesis that absolute numbers of cortical neurons, particularly in the associative areas, are the most direct predictor of cognitive capabilities (Herculano‐Houzel 2017). We previously raised the suspicion that these high estimates of numbers of cortical neurons in cetaceans were due to undersampling (Kazu et al. 2014; and see Discussion). To settle such a crucial issue in brain evolution and comparative cognition, we needed to apply the isotropic fractionator cell counting method to cetacean brains, a method that has been applied to over 200 vertebrate species with remarkably consistent results (Herculano‐Houzel 2022).

Here, we estimate the cellular composition of the brain of a northern minke whale (*Balaenoptera acutorostrata*), a small mysticete, with an average body length of 8 m, an average body mass of 6.76 tons, and a brain more than twice the mass of the human brain (Armstrong and Siegfried 1991; Markussen et al. 1992; Tamura and Konishi 2006). We employed the isotropic fractionator technique (Herculano‐Houzel and Lent 2005) that allows for rapid determination of the numbers of cells within any dissectable tissue without incurring bias from tissue volume. Our aims are to determine: (1) Whether the northern minke whale brain studied and its substructures, especially the cerebral cortex, share the same neuronal scaling rules that apply to artiodactyls and other non‐primate mammals, or if the northern minke whale has diverged from these scaling rules, as suggested by the stereological estimate of 13 billion neurons in its cerebral cortex (Eriksen and Pakkenberg 2007); and (2) whether the northern minke whale follows the same non‐neuronal scaling rule that applies to all therians examined to date, or has deviated from this rule.

## 2 Materials and Methods

### 2.1 Specimen

We examined the right half of the brain of one adult male northern minke whale (*B. acutorostrata*), which was acquired during the whaling season in Iceland in partnership with the Iceland Ministry of Fisheries and Agriculture. The brain was obtained opportunistically after the death of the animal according to Icelandic commercial whaling practices. The specimen was treated and used according to the guidelines of the University of Witwatersrand Animal Ethics Committee, which correspond with those of the National Institutes of Health (NIH) for the care and use of animals in scientific experimentation (Dell, Karlsson, et al. 2016). The brain was removed from the skull within 1 h of death and immersion fixed in 4% paraformaldehyde in 0.1 M phosphate buffer (PB) for 72 h at 4°C. The brain was then cryoprotected in 30% sucrose in 0.1 M PB for 14 days and subsequently stored in antifreeze solution at −20°C until processed (Manger et al. 2009).

### 2.2 Dissection

**FIGURE 1:**
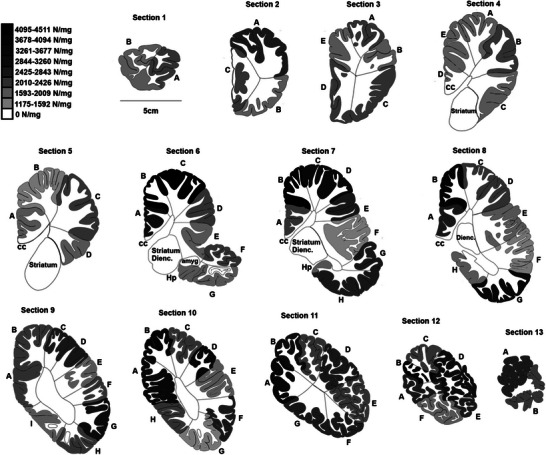
Diagram of the 13 coronal sections of the right cerebrum of the minke whale brain analyzed, each 12.8 mm thick, numbered from anterior (Section 1) to posterior (Section 13), and their 148 cortical subdivisions. Each block indicated by the letters represents a piece of gray matter and corresponding subcortical white matter processed separately. The color intensity of each subdivision indicates the neuronal density according to the grey scale bar on the left. White matter is not colored due to its low neuronal density. Amyg, amygdala; cc, corpus callosum; Dienc, diencephalon; Hp, hippocampus.

The entire right half of the brain was subjected to analysis using the isotropic fractionator by dissecting it into its main structures and subdividing each into smaller blocks of tissue amenable to homogenization in a handheld Tenbroeck glass homogenizer. First, the cerebellum (CB) was separated by cutting the cerebellar peduncle at the surface of the brainstem, then it was sectioned manually into 5 sagittal sections of 12.8 mm thickness. The brainstem was dissected by cutting through the plane anterior to the superior colliculi and posterior to the diencephalon and hypothalamus. Once dissected, the brainstem was further separated into mesencephalon, pons (rostral hindbrain), and medulla (caudal hindbrain), which were further subdivided for processing. The remaining cerebrum was sectioned manually into 13 coronal sections of 12.8 mm each. From each section, the diencephalon + corpus striatum, the amygdala, and the hippocampus were separated. The remaining cerebral cortex was further systematically dissected into smaller parts (lettered in Figure 1) and separated into gray and white matter, providing a total of 148 tissue blocks of cerebral cortex that were each processed and counted separately. No part of the brain hemisphere was left uncounted. The left half of the brain was preserved for histological studies.

### 2.3 Isotropic Fractionator

The total number of cells, neurons, and non‐neuronal cells (the majority of which are presumably glial cells, but include capillary cells; Ventura‐Antunes et al. 2022) was estimated using the isotropic fractionator method (Herculano‐Houzel and Lent 2005). After dissection, the tissue blocks were weighed and subsequently mechanically dissociated in a detergent solution of 1% Triton X‐100 in 40 mM trisodium citrate using a Tenbroeck glass homogenizer. The homogenate and several washes of the homogenizer were transferred to a graduated cylinder using a glass pipette. To visualize the nuclei, the fluorescent DNA marker DAPI (4,6‐diamidino‐2‐phenylindoledihydrochloride, Invitrogen, Carlsbad, Calif., USA) was added to the suspension from a stock solution at 20 mg/L (dilution 1:20–1:50), and the final volume of the suspension was recorded. Before cell counting, the suspension was agitated to ensure homogeneity. Four aliquots of the homogenous suspension were counted in an improved Neubauer chamber under a fluorescent microscope (Zeiss, Jena, Germany). In each aliquot, the number of nuclei was counted in either 40 or 100 nL within the counting frame of the chamber, whichever sufficed to ensure that at least 60 and not more than 300 nuclei were counted per aliquot. The average number of nuclei per milliliter in the samples, as well as the coefficient of variation (CV = standard deviation/average), were then determined for each sample. CVs were consistently below 0.15, and typically below 0.10. The total number of cells in the suspension was then obtained by multiplying the average number of nuclei per milliliter by the total volume of the suspension. Thus, the isotropic fractionation provides estimates of cell numbers that are as reliable as those obtained with rigorous stereology (Herculano‐Houzel, Kaas, et al. 2015).

To determine the number of neurons in each block of brain homogenized, a sample of 500 µL of each suspension was washed with PBS (0.1 M) and incubated overnight in the dark at 4°C with Cy3‐labeled rabbit polyclonal neuronal nuclear antigen antibody (1:150 dilution; NeuN, RRID:AB_11204707). The following day the samples were washed and resuspended in PBS. The percentage of nuclei that belonged to neurons was determined by counting at least 500 DAPI‐stained nuclei and establishing the fraction that was also NeuN positive. The total number of neurons in each sample was determined by multiplying the total number of cells in the structure by the NeuN‐positive fraction obtained. The total number of non‐neuronal cells was determined by subtracting the total number of neurons from the total number of cells. Numbers of cells composing each major brain structure were obtained from the sum of all tissue blocks of the region. Densities of neurons and non‐neurons (cells/mg) in each structure were then determined by dividing the number of neurons or non‐neurons by the mass (mg) of the structure.

### 2.4 Data Analysis

In order to compare our results with previously reported multi‐species mammalian datasets (Herculano‐Houzel, Catania, et al. 2015; Dos Santos et al. 2017; Jardim‐Messeder et al. 2017), the values for the cerebral cortex include the entorhinal cortex, the piriform cortex, the amygdala, and the hippocampus, although we also report those values separately here. Similarly, the pons, medulla, mesencephalon, and diencephalon plus corpus striatum, although analyzed and reported separately, are combined here as “rest of brain” (ROB; Table 1). The whole brain is the sum of the cerebral cortex, CB, and ROB. All values reported correspond to the numbers obtained for the right half of the brain multiplied by two in order to be able to compare to previously published data (Herculano‐Houzel, Catania, et al. 2015). This approach does not consider any differences between the two halves of the brain, since it can be assumed that any asymmetries that might occur are negligible, for the purposes of our analysis, compared to the differences across species that span several orders of magnitude (Herculano‐Houzel, Catania, et al. 2015).

**TABLE 1:** Summary of the cellular composition of the major brain structures of the northern minke whale. All numbers refer to the entire brain, that is, our estimates for the right hemisphere multiplied by 2. Whole brain consists of cerebral cortex, cerebellum, and rest of brain; the other lines in the table list the hippocampus, amygdala, and subdivisions of the ROB separately.

Structure	Mass, g	Neurons	Non‐neuronal cells	Neurons/mg	Non‐neuronal cells/mg	Non‐neuronal cells/neuron
Whole brain	2,683.9	57,472,789,206	173,396,134,794	21,414	64,505	3.0
Cerebral cortex (including Hp + amygdala)	2,052.2	3,166,399,674	133,472,299,326	1,543	65,038	42.2
Cerebral cortical grey matter	1,136.5	2,690,224,290	47,471,362,210	2,367	41,768	17.6
Cerebral cortical white matter	900.5	458,756,249	85,379,606,251	509	94,816	186.1
Cerebellum	415.9	54,216,171,588	22,313,190,913	130,353	53,648	0.4
Rest of brain (diencephalon+striatum, mesencephalon, pons and medulla)	215.8	88,149,444	17,317,213,056	409	80,256	196.4
Amygdala	7.5	4,137,000	586,863,000	551	78,144	141.9
Hippocampus	7.7	13,282,136	625,467,864	1 725	81,208	47.1
Diencephalon + Striatum	123.3	70,703,706	11,085,646,296	573	89,908	156.9
Mesencephalon	29.7	5,413,766	2,305,861,236	182	77,638	426.6
Pons	33.7	6,321,406	2,096,603,596	188	62,214	330.9
Medulla oblongata	29.1	5,710,568	1,829,101,932	196	62,856	320.7

Data analysis was performed with JMP 14.0 software (SAS, USA). Least‐squares regressions of log‐transformed data to linear functions were performed to find the power function that best fit each distribution. All allometric exponents are reported ± standard error (SE) and with the corresponding *p* value. To test whether the northern minke whale studied conforms to the previously determined scaling rules for different brain structures in relation to numbers of cells and structure mass, the values for the northern minke whale were compared to those predicted for a hypothetical artiodactyl or non‐primate brain according to the scaling rules obtained for previously reported mammalian species (Herculano‐Houzel, Catania, et al. 2015; Dos Santos et al. 2017; Jardim‐Messeder et al. 2017).

## 3 Results

### 3.1 Mass and Numbers of Neurons in the Minke Whale Brain

The whole northern minke whale brain analyzed in the current study had a total mass of 2683.9 g and was comprised of a total of 57.5 × 10^9^ neurons (Table 1). Although the cerebral cortex, at 2052.2 g, amounts to 76.5% of the mass of the whole brain, its 3.17 × 10^9^ neurons correspond to only 5.5% of the total number of brain neurons. Conversely, while the CB had a mass of 415.9 g, representing 15.5% of the northern minke whale brain mass, it contained 54.2 × 10^9^ neurons, or 94.3% of the total number of brain neurons. The remaining brain structures, which we refer to as rest of brain or ROB (which includes the subpallial telencephalon, diencephalon and brainstem, but excludes the cerebral cortex and CB), had a mass of 215.8 g (8.0% of brain mass) and was comprised of 88.15 × 10^6^ neurons, or 0.15% of the total number of brain neurons (Table 1). Within the cerebral cortex, the hippocampus had a mass of 7.7 g and was composed of 13.3 × 10^6^ neurons, or 0.4% of all cortical neurons, in line with its mass of 0.4% of the whole cerebral cortex.

### 3.2 Minke Whale Numbers of Neurons Compared to Other Mammals

#### 3.2.1 The Whole Brain

**FIGURE 2:**
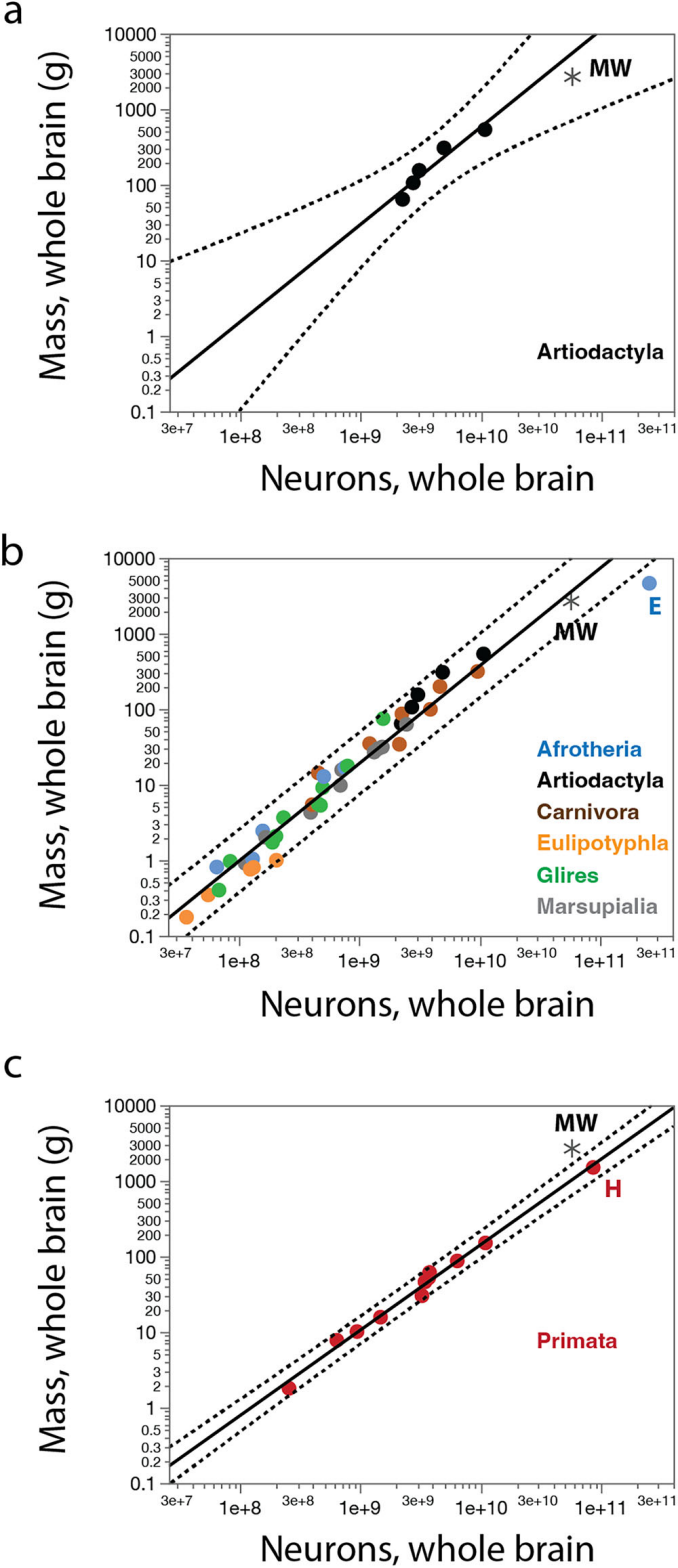
When regressing brain mass against total numbers of neurons in the brain across mammalian species, we find that the northern minke whale brain (black asterisk) studied has as many neurons as expected for a hypothetical artiodactyl (**a**) or non‐primate (**b**) brain of the same mass, falling within the 95% prediction intervals (dotted lines); but has substantially fewer neurons than expected for a hypothetical primate brain of the same mass (**c**). **E**, African elephant; **H**, human; **MW**, minke whale. Plotted functions are (a) M_br_ = e^−23.284^ N_br_^1.288 ± 0.215^ (without the minke whale), (b) M_br_ = e^−23.795^ N_br_^1.291 ± 0.041^ (without the minke whale), and (c) M_br_ = e^−21.061^ N_br_^1.130 ± 0.036^.

Across artiodactyls, we previously found that brain mass scales with total numbers of brain neurons as a power function of exponent of 1.288 ± 0.215 (Kazu et al. 2014; Figure 2a) that is very similar to the exponent of 1.291 ± 0.041 that applies across a range of non‐primate mammals (Artiodactyla, Afrotheria, Carnivora, Eulipotyphla, Marsupialia and Glires; Kazu et al. 2014; Herculano‐Houzel, Manger, et al. 2014; Herculano‐Houzel, Catania, et al. 2015; Dos Santos et al. 2017; Jardim‐Messeder et al. 2017; Figure 2b). Solving the equations for a non‐primate brain mass of 2683.9 g yields a predicted 38.7 billion neurons in the brain, which is less than the observed 57.5 billion, but well within the 95% prediction intervals for both artiodactyls alone and for non‐primate species as a whole (asterisk in Figure 2b). In contrast, the data for the northern minke whale falls above the 95% prediction interval for the relationship between brain mass and numbers of brain neurons in primates (Herculano‐Houzel et al. 2007; Azevedo et al. 2009; Gabi et al. 2010), which has an exponent of 1.130 ± 0.036 (Figure 2c). These findings indicate that the northern minke whale brain has substantially fewer neurons than expected for its brain mass compared to a hypothetical primate of a similar brain mass, but approximately the number of brain neurons expected for a non‐primate mammal of its brain mass, and for a cetartiodactyl in particular.

#### 3.2.2 The Cerebral Cortex

**FIGURE 3:**
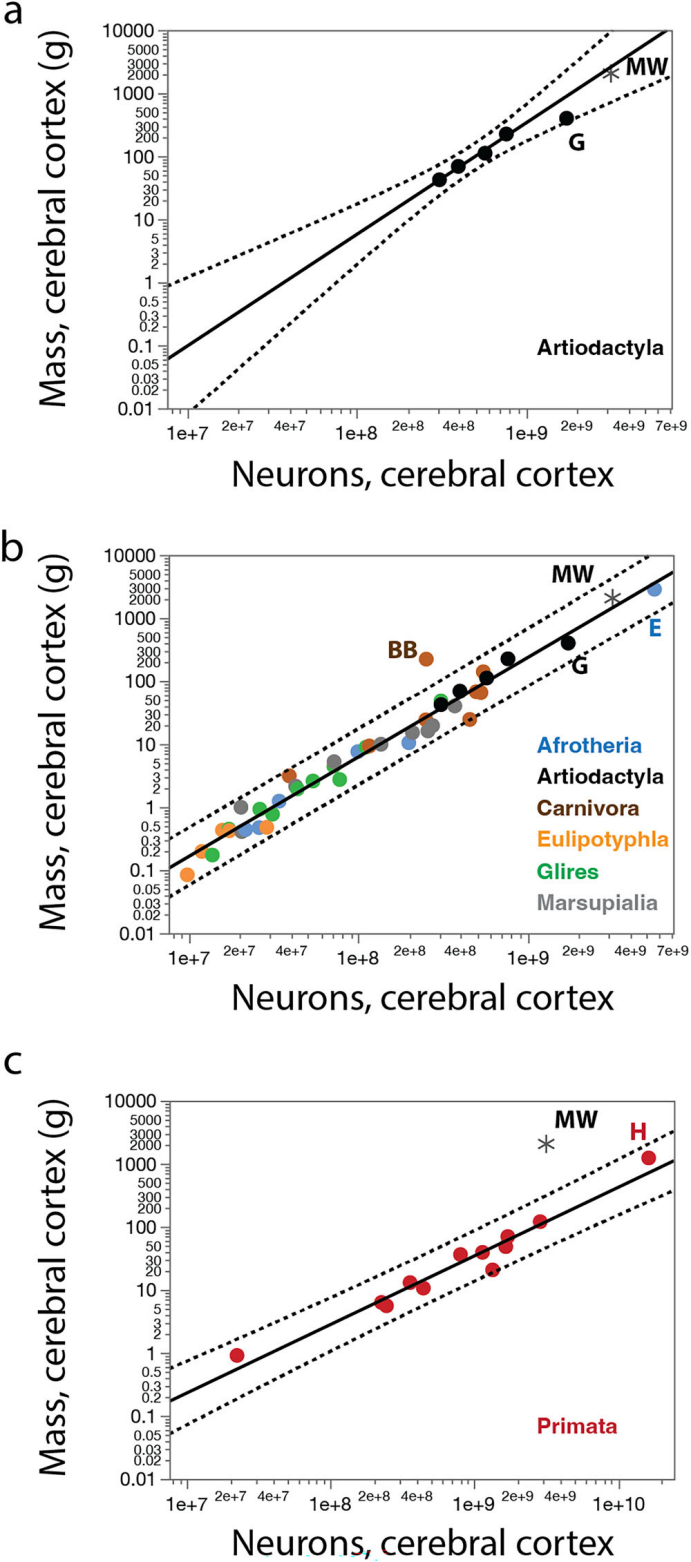
When regressing cerebral cortical mass against total numbers of cerebral cortical neurons across mammalian species, we find that the Northern minke whale brain (black asterisk) studied has as many cortical neurons as expected for a hypothetical artiodactyl (**a**) or non‐primate (**B**) cerebral cortex of the same mass, falling within the 95% prediction intervals (dotted lines); but has substantially fewer neurons than expected for a hypothetical primate cerebral cortex of the same mass (**C**). **BB**, brown bear; **E**, African elephant; **G**, giraffe (juvenile); **H**, human; **MW**, minke whale. Plotted functions are (**a**) *M*_cx_ = *e* ^−30.834^ *N*_cx_^1.769 ± 0.147^ (without the giraffe or minke whale), (**b**) *M*_cx_ = *e* ^−27.206^ *N*_cx_^1.576 ± 0.052^ (without the minke whale), and (**c**) *M*_cx_ = e^−18.987^ *N*_cx_^1.087 ± 0.074^.

The artiodactyl cerebral cortex gains mass as a power function of its total number of cortical neurons with an exponent of 1.769 ± 0.147 (excluding the juvenile giraffe; Kazu et al. 2014, Figure 3a), which is similar to the exponent of 1.576 ± 0.052 that applies to non‐primate mammalian species (Figure 3b). The latter relationship predicts that a non‐primate cerebral cortex of the size of the northern minke whale cortex studied should contain 3.4 billion neurons, which is an excellent match to the 3.2 billion neurons we found (Table 1), well within the 95% prediction intervals determined for a hypothetical artiodactyl or non‐primate cerebral cortex of a similar mass. In contrast, the data for the northern minke whale falls substantially outside the 95% prediction interval of the relationship that describes the scaling of cerebral cortical mass against cerebral cortical neuronal numbers across primate species (Herculano‐Houzel et al. 2007; Azevedo et al. 2009. Gabi et al. 2010; Figure 3c), which predicts for a cerebral cortex of 2052.2 g a total of 35.9 billion neurons, more than ten times higher than the 3.2 billion we found in the minke whale cortex.

**FIGURE 4:**
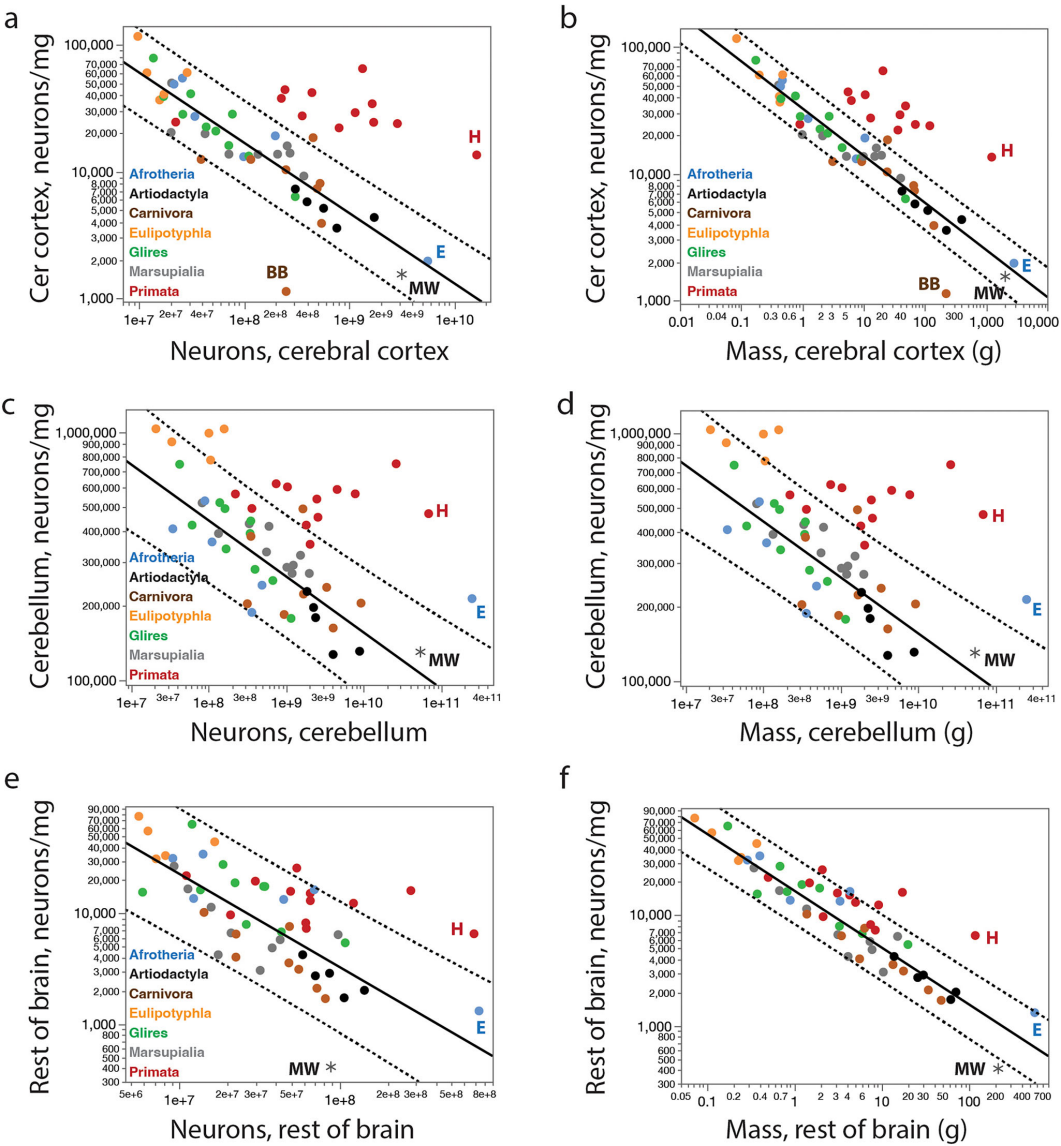
Neuronal densities in the minke whale brain are as low as expected in the cerebral cortex and cerebellum, but extraordinarily low in the rest of brain. (**a, b**) Neuronal density in the combined grey and white matter of the cerebral cortex of non‐primate species (excluding the minke whale and the brown bear) decreases as a power function of numbers of cortical neurons of exponent −0.555 ± 0.039 (**a**; *r* ^2^ = 0.836, *p* < 0.0001), and as a power function of cortical mass with exponent −0.372 ± 0.016 (**b**; *r* ^2^ = 0.934, *p* < 0.0001). (**c, d**) Neuronal density in the cerebellum of non‐primate, non‐eulipotyphlan species (excluding the minke whale and the elephant) decreases as a power function of numbers of cerebellar neurons of exponent −0.226 ± 0.032 (**c**; *r* ^2^ = 0.587, *p* < 0.0001), and as a power function of cerebellar mass with exponent −0.204 ± 0.021 (**d**; *r* ^2^ = 0.732 ± 0.021). Notice that the elephant cerebellum has a significantly higher neuronal density than expected for both its mass and number of neurons, but the minke whale cerebellum has the density expected for a generic artiodactyl, or even non‐primate, cerebellum. (**e, f**) Neuronal density in the ROB of non‐primate species (excluding the minke whale) decreases as a power function of numbers of neurons in the ROB with exponent −0.843 ± 0.099 (**e**; *r* ^2^ = 0.636, *p* < 0.0001), and as a power function of ROB mass with exponent −0.515 ± 0.026 (**f**; *r* ^2^ = 0.904, *p* < 0.0001). Notice that in both cases the minke whale has a lower neuronal density in the ROB than expected for a generic artiodactyl or non‐primate species.

We find an average of only 1543 neurons/mg in the combined grey and white matter of the minke whale cerebral cortex (Table 1). While this is a very low neuronal density, it falls within the prediction interval for non‐primate species given the steep negative scaling of neuronal density with increasing numbers of neurons in the cerebral cortex (Figure 4a) or increasing cortical mass (Figure 4b). Such a low neuronal density in the minke whale cortex is therefore to be expected for an artiodactyl or non‐primate cerebral cortex of its size and number of neurons.

**FIGURE 5:**
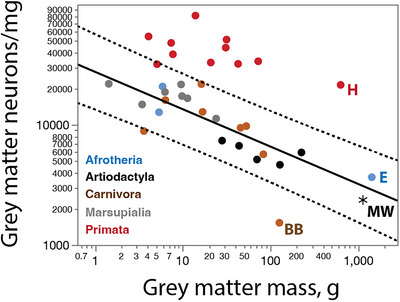
Neuronal densities in the grey matter of the minke whale cerebral cortex are as low as expected for the mass of the cortical grey matter, conforming to the power function that applies across non‐primate species with available data in the dataset (excluding the brown bear; exponent −0.311 ± 0.038 (*r* ^2^ = 0.759, *p* < 0.0001). Notice that neuronal densities of 10,000 neurons/mg and above for cortical grey matter values above 400 g would be exceptionally high for non‐primate species, and match instead the neuronal density found in the human cortical grey matter.

The relatively large mass of white matter included in the calculation of neuronal density, shown in Figure 4a,b obviously contributes to the low values we report for the whole minke whale cortex. This remains an important analysis, though, for it tests the minke whale cerebral cortex for conformity to the scaling laws that we have previously found to apply to cetartiodactyls specifically, and to non‐primate species generally. Because we dissected and processed grey and white matter separately, we could estimate that the grey matter alone, at 1136.5 g and 2.7 billion neurons, has an overall average neuronal density of 2367 neurons/mg (Table 1). As shown in Figure 5, this density places the minke whale we analyzed squarely in the 95% prediction interval for non‐primate species. A total of 458.8 million neurons were found in the 900.5 g subcortical white matter, at an average density of 509 neurons/mg. While a number of these are likely to be due to contamination of the dissected white matter with the lowest grey matter layers, densities of at least 100 neurons/mg were a constant finding even in cores of subcortical white matter free of any grey matter contamination.

#### 3.2.3 The Cerebellum

**FIGURE 6:**
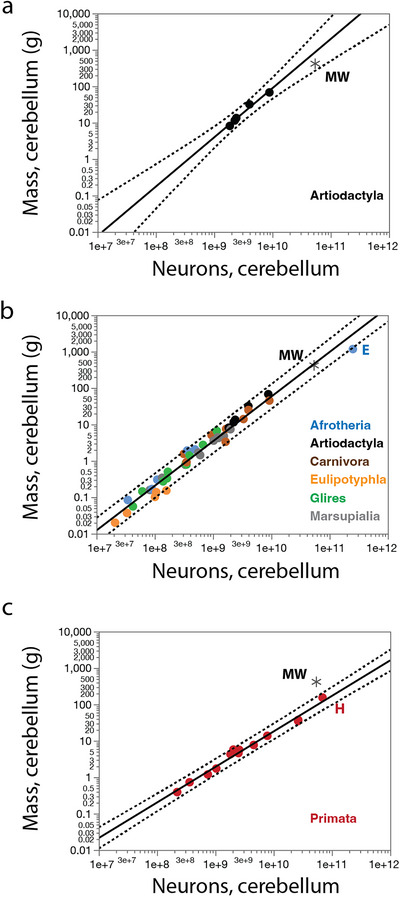
When regressing cerebellar mass against total numbers of cerebellar neurons across mammalian species, we find that the Northern minke whale brain (black asterisk) studied has the number of cerebellar neurons expected for a hypothetical artiodactyl (**a**) or non‐primate (**b**) cerebellum of the same mass, falling within the 95% prediction intervals (dotted lines), but has substantially fewer neurons than expected for a hypothetical primate cerebellum of the same mass (**c**). **BB**, brown bear; **E**, African elephant; **G**, giraffe (juvenile); **H**, human; **MW**, minke whale. Plotted functions are (**a**) *M*_cb_ = *e* ^−26.632^ *N*_cb_^1.351 ± 0.118^ (without the minke whale), (**b**) *M*_cb_ = *e* ^−24.151^ *N*_cb_^1.225 ± 0.031^ (without the minke whale), and (**c**) *M*_cb_ = *e* ^−19.572^ *N*_cb_^0.976 ± 0.036^.

The artiodactyl CB gains mass as a power function of its total number of neurons with an exponent of 1.351 ± 0.118 (Figure 6a), which is similar to the exponent of 1.225 ± 0.031 that applies to the CB of non‐primate mammalian species (Figure 6b). In both cases, the number of cerebellar neurons in the northern minke whale studied was well within the 95% prediction interval calculated for a generic artiodactyl or non‐primate mammal of its cerebellar mass. In contrast, the data for the minke whale fall substantially outside the 95% prediction interval of the relationship that describes the scaling of cerebellar mass against numbers of cerebellar neurons across primate species (Figure 6c).

We find in the minke whale CB an average of 130,353 neurons/mg (Table 1). While this is a very low neuronal density, it falls within the prediction interval for non‐primate species given the steep negative scaling of neuronal density with increasing numbers of neurons in the CB (Figure 4c) or increasing cerebellar mass (Figure 4d). The fairly low neuronal density in the minke whale CB is what is to be expected for an artiodactyl or non‐primate CB of its size and number of neurons.

### 3.3 Rest of Brain

**FIGURE 7:**
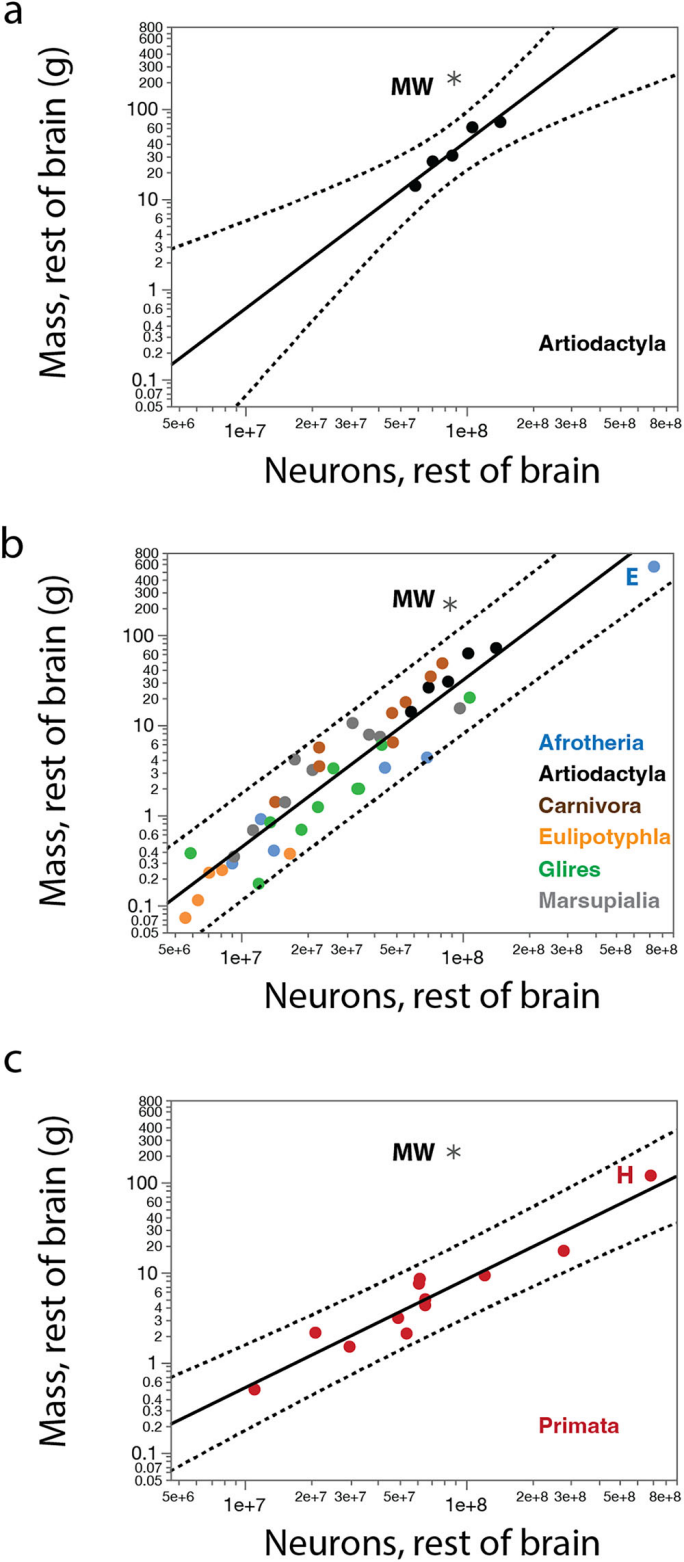
The ROB of the northern minke whale brain studied (black asterisk), that is, the ensemble of non‐cerebral cortical, non‐cerebellar structures, has significantly fewer neurons than expected for a generic artiodactyl (**a**), non‐primate (**b**), or primate (**c**) ROB of the same mass, falling outside the 95% prediction intervals (dotted lines). **E**, African elephant; **H**, human; **MW**, minke whale. Plotted functions are (**a**) *M*_rob_ = *e* ^−30.323^ *N*_rob_^1.850 ± 0.303^ (without the minke whale), (**b**) *M*_rob_ = *e* ^−30.562^ *N*_rob_^1.845 ± 0.100^ (without the minke whale), and (**c**) *M*_rob_ = *e* ^−19.962^ *N*_rob_^1.198 ± 0.116^.

Surprisingly, we find that the minke whale ROB has a significantly smaller ROB mass than expected for its number of neurons, which can be interpreted equally as significantly fewer neurons than expected for a generic artiodactyl or non‐primate mammal of similar ROB mass. Figure 6 shows that the northern minke whale ROB is excluded from the 95% prediction interval of the scaling functions that relate ROB mass to the number of neurons in artiodactyls (Figure 7a), non‐primate mammals as a whole (Figure 7b), as well as primates (Figure 7c).

Accordingly, we find that the overall neuronal density within the ROB of the northern minke whale studied is significantly lower than would be predicted for a generic non‐primate mammal of similar ROB neuronal number (Figure 4e) or ROB mass (Figure 4f). The northern minke whale, with an average neuronal density of only 445 neurons/mg, or about 1/3 the expected for ROB mass, is, to date, the mammalian species with the lowest average neuronal density observed in the ROB, well below the 95% prediction interval for non‐primate mammalian species.

To determine whether it is the mass of the ROB or the number of neurons in the ROB of the minke whale that is the outlier, we analyzed how these variables scale according to body mass and how they scale with the corresponding variables in other brain structures.

### 3.4 Scaling of Brain Mass and Numbers of Neurons With Body Mass

**FIGURE 8:**
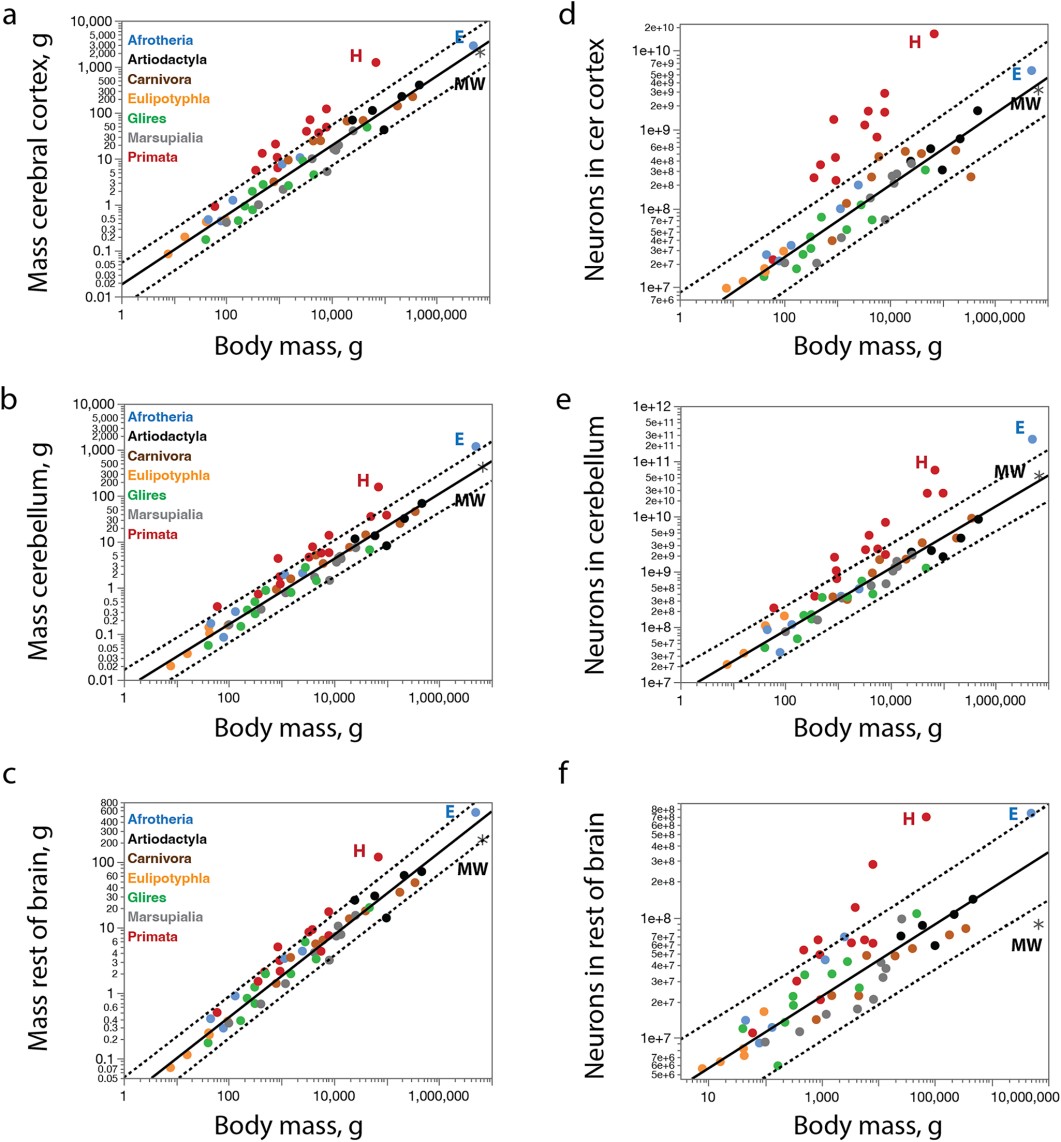
The northern minke whale brain studied has the mass and number of neurons in the cerebral cortex and cerebellum expected for a non‐primate mammalian species of its body mass, but has significantly less mass and fewer neurons in the ROB than expected. Plotted power functions (solid lines) and 95% prediction interval (dotted lines) apply to non‐primate species in the dataset other than the minke whale. Functions are (**a**) *M*_cx_ = *e* ^−4.019 ± 0.205^ *M*_bd_^0.756 ± 0.024^, (**b**) *M*_cb_ = *e* ^−5.090 ± 0.708^ *M*_bd_^0.708 ± 0.022^, (**c**) *M*_rob_ = *e* ^−3.765 ± 0.147^ *M*_bd_^0.628 ± 0.018^, (**d**) *N*_cx_ = e^14.903 ± 0.199^ *M*_bd_^0.455 ± 0.024^, (**e**) *N*_cb_ = e^15.694 ± 0.201^ *M*_bd_^0.561 ± 0.024^, and (**f**) *N*_rob_ = *e* ^14.847 ± 0.172^ *M*_bd_^0.299 ± 0.020^. **E**, African elephant; **H**, human; **MW**, minke whale.

The minke whale studied had a cerebral cortex and CB of the mass expected for a non‐primate mammal of its body mass (Figure 8a,b). The mass of the ROB, however, was just outside of the 95% prediction interval, being small for the size of the body (Figure 8c). Similarly, we found that the minke whale cerebral cortex and CB had the numbers of neurons expected for a generic non‐primate mammal of this species’ body mass (Figure 8d,e), but significantly fewer neurons than expected in the ROB (Figure 8f).

**FIGURE 9:**
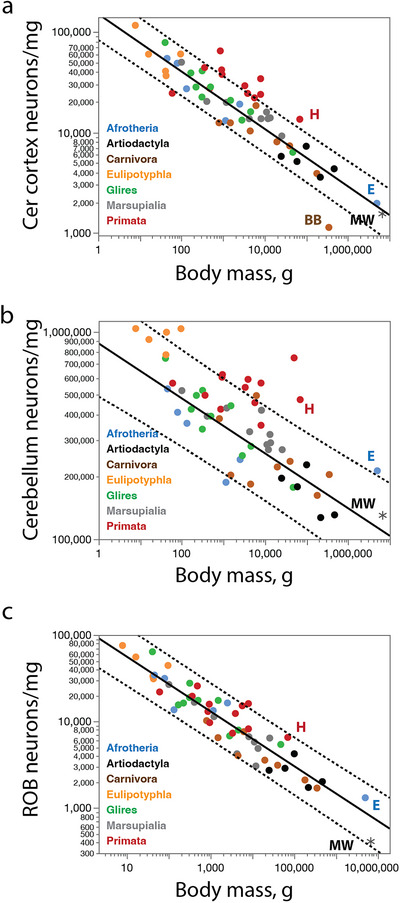
The northern minke whale brain studied has, in all brain divisions, the neuronal density expected for a non‐primate mammalian species of its body mass. (**a**) Power function plotted for non‐primate species (excluding the brown bear and the minke whale): *DN*_cx_ = *e* ^11.927 ± 0.114^ *M*_bd_^−0.286 ± 0.014^. (**b**) Power function plotted for non‐primate species (excluding the brown bear and the minke whale): *DN*_cb_ = *e* ^13.688 ± 0.140^ *M*_bd_^−0.133 ± 0.016^, (**c**) *DN*_rob_ = *e* ^11.664 ± 0.145^ *M*_bd_^−0.316 ± 0.018^. The minke whale data point falls within all 95% prediction intervals (dotted lines). **E**, African elephant; **H**, human; **MW**, minke whale.

Interestingly, despite the smaller mass and fewer neurons in the ROB than predicted for its body mass, the density of neurons in the ROB still fell within the 95% prediction interval of the function that describes the concerted decrease with increasing body mass that we have previously reported to apply across all mammalian species, including primates, although on the low end of the interval (Herculano‐Houzel, Catania, et al. 2015), as shown in Figure 9. The minke whale thus seems to have both a smaller ROB and fewer ROB neurons than expected for its body mass, but in a coordinated manner such that the expected neuronal density for its body mass is still somewhat preserved.

### 3.5 Scaling of Mass and Numbers of Neurons Between the Major Divisions of the Brain

**FIGURE 10:**
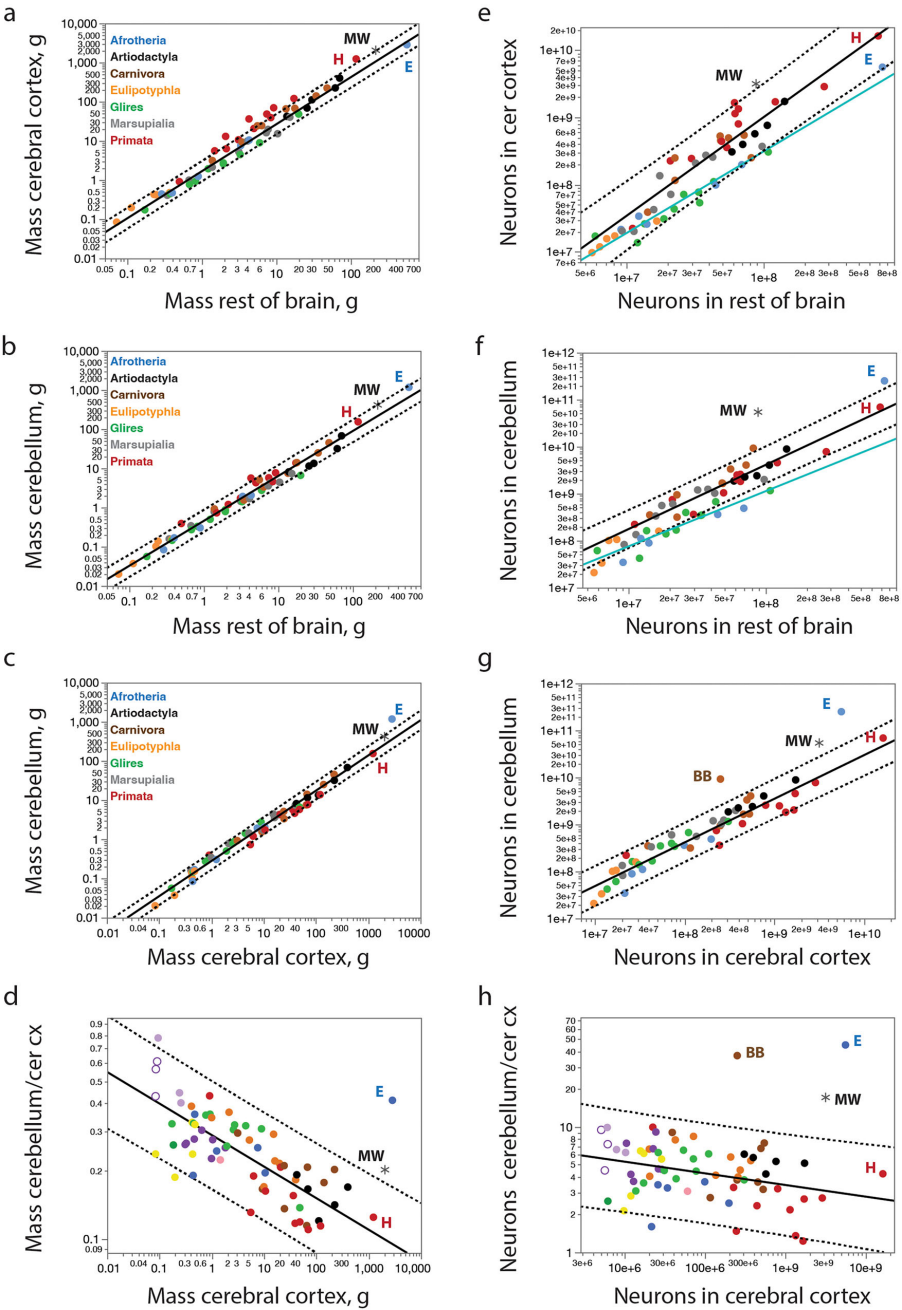
**The minke whale has a small ROB and an overly large cerebellum for the mass of its cerebral cortex (a–d), with fewer ROB neurons and many more cerebellar neurons than expected for the number of neurons in the cerebral cortex (e–h)**. Functions are (**a**) *M*_cx_ = *e* ^0.514 ± 0.051^ *M*_rob_^1.201 ± 0.022^ (excluding primates and the minke whale); (**b**) *M*_cb_ = *e* ^−0.832 ± 0.041^ *M*_rob_^1.095 ± 0.020^ (excluding primates, the African elephant and the minke whale); (**c**) *M*_rob_ = *e* ^−1.281 ± 0.048^ *M*_rob_^0.898 ± 0.017^ (excluding primates, the African elephant and the minke whale); (**d**) *M*_cb/cx_ = *e* ^−1.252 ± 0.038^ *M*_cx_^−0.137 ± 0.014^ (excluding the African elephant and the minke whale); (**e**) *N*_cx_ = *e* ^−2.748 ± 0.903^ *N*_rob_^1.211 ± 0.053^ (eulipotyphlans, glires and all afrotheria, plotted in acqua) and *N*_cx_ = *e* ^−6.230 ± 1.828^ *N*_rob_^1.464 ± 0.103^ (artiodactyls, excluding the minke whale, plus carnivorans, marsupials, and primates, plotted in black); (**f**) *N*_cb_ = *e* ^−0.706 ± 1.992^ *N*_rob_^1.170 ± 0.119^ (eulipotyphlans, glires and afrotheria minus the African elephant, plotted in acqua) and *N*_cb_ = *e* ^−2.952 ± 1.433^ *N*_rob_^1.362 ± 0.081^ (artiodactyls, excluding the minke whale, plus carnivorans, marsupials, and primates, plotted in black); (**g**) *N*_cb_ = *e* ^2.733 ± 0.735^ *N*_cx_^0.929 ± 0.039^ (all species minus the brown bear, African elephant, and minke whale); (**h**) *N*_cb/cx_ = *e* ^3.157 ± 0.557^ *N*_cx_^−0.093 ± 0.030^ (excluding the African elephant, the brown bear and the minke whale). **E**, African elephant; **H**, human; **MW**, minke whale.

Compared with other non‐primate mammalian species, the minke whale brain we analyzed had larger cerebral cortical and cerebellar mass than expected for its ROB mass (Figure 10a,b), which is consistent with our finding that the two former structures have the mass expected for the mass of the minke whale body, while the ROB is significantly smaller than predicted (Figure 8a,b). Relative to each other, however, the CB and cerebral cortex of the minke whale examined appear at first glance to have the structural mass expected for other mammalian species (Figure 10c), with a relative cerebellar mass of 15.5% of brain mass. In the latter case, the African elephant is the known outlier, with an exceptionally large CB for its cerebral cortical mass that reaches 25.4% of brain mass (Maseko et al. 2012; Herculano‐Houzel, Avelino‐de‐Souza, et al. 2014). However, closer examination of the proportional size of the CB relative to the cerebral cortex reveals that the CB of the minke whale is indeed exceptionally large compared to all non‐primate mammals (Figure 10d).

Numbers of neurons in the cerebral cortex scale differently with numbers of neurons in the ROB across the ensemble of artiodactyls, carnivorans, Asian marsupials, and primates (including humans), in comparison to the ensemble of afrotherians (including the elephant), eulipotyphlans, and glires (Figure 10e; Herculano‐Houzel, Manger, et al. 2014). Although, the minke whale has a cortical mass that is still not significantly different from the mass predicted for its ROB mass (Figure 10a), it has fewer than 1/3 the neurons in its ROB expected for its number of cortical neurons (Figure 10e). Similarly, while the minke whale has a cerebellar mass that falls within the mass interval predicted for its ROB mass (Figure 10b), it has about 13.5 times as many cerebellar neurons as predicted for its number of ROB neurons (Figure 10f). While both these results indicate an excess of cortical and cerebellar neurons in the minke whale over the numbers predicted for their numbers of ROB neurons, it could still be the case that the apparent “excess” is actually due to a smaller than predicted number of ROB neurons for the minke whale body mass, given that its numbers of cortical and cerebellar neurons fall well within the prediction interval for body mass (Figure 8).

Strikingly, however, we find that the minke whale is, together with the brown bear (Jardim‐Messeder et al. 2017) and the African elephant (Herculano‐Houzel, Avelino‐de‐Souza, et al. 2014), an outlier in the proportion between numbers of neurons in the CB and cerebral cortex (Figure 10g). We have shown previously that most mammals share a consistent ∼4:1 ratio of cerebellar to cortical neurons (Herculano‐Houzel, Manger, et al. 2014), with the only known exceptions so far bring the African elephant, with a 45:1 ratio (Herculano‐Houzel, Avelino‐de‐Souza, et al. 2014) and the brown bear, with 37:1 (Jardim‐Messeder et al. 2017). The northern minke whale analyzed herein adds a third exception, with 17.1 neurons in the CB for every neuron in the cerebral cortex (Figure 10h). This places the data for the northern minke whale above the 95% prediction interval calculated from regressing the numbers of neurons in the CB against number of cortical neurons across mammals, including primates (Figure 10g), which describes a power relationship that has an exponent of 0.929 ± 0.039 (*r* ^2^ = 0.919), indistinguishable from linearity, and indeed better described as a linear function of slope 4.15 ± 0.08 (*r* ^2^ = 0.984; not shown).

### 3.6 Numbers and Densities of Non‐Neuronal Cells in the Minke Whale Brain

**FIGURE 11:**
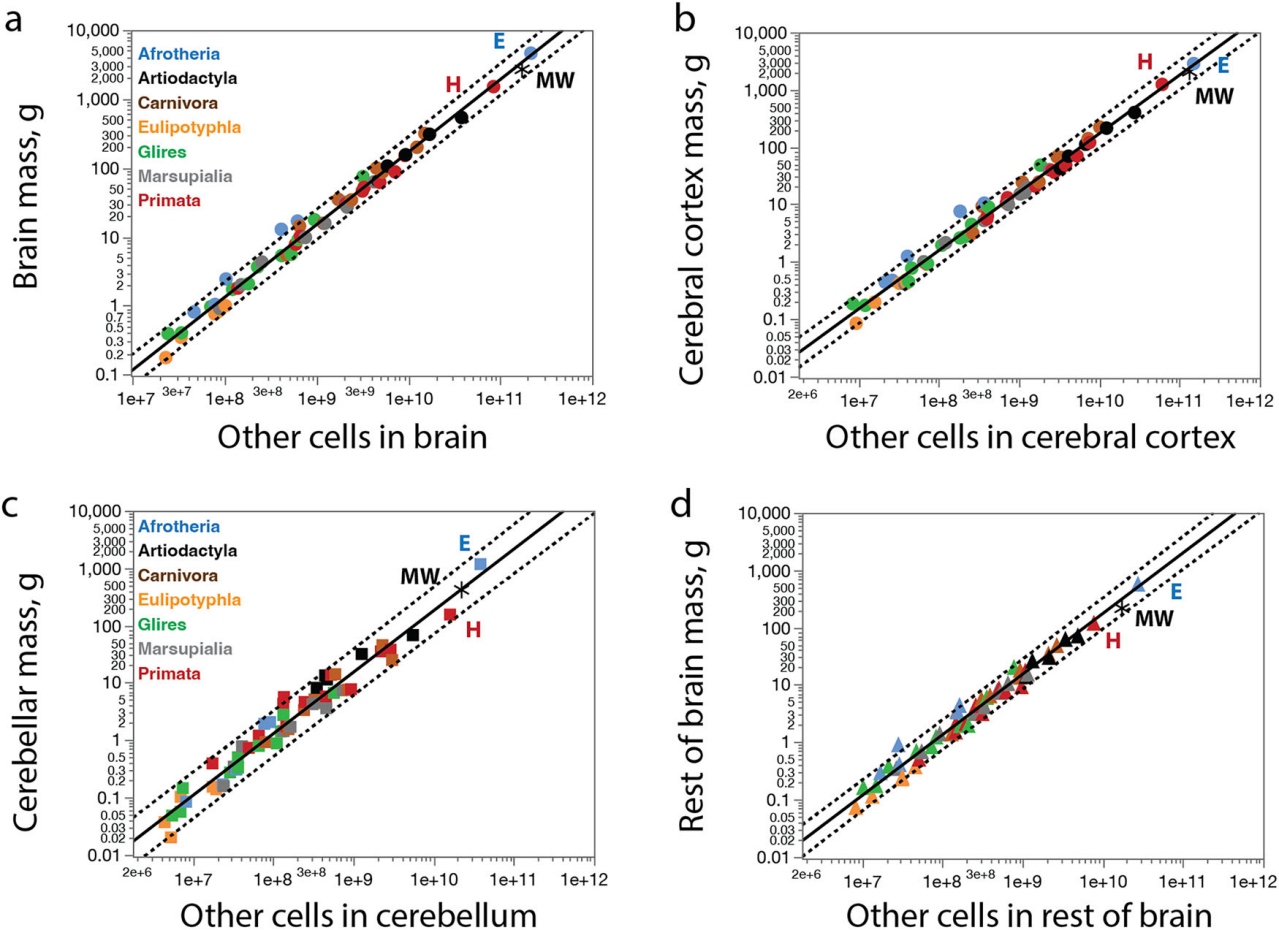
**The minke whale brain has the number of non‐neuronal cells expected for a generic mammalian brain in each of its main divisions**. Functions plotted include all mammalian species in the dataset, minus the minke whale, and are (**a**) *M*_br_ = *e* ^−19.119 ± 0.332^ *O*_br_^1.053 ± 0.016^; (**b**) *M*_cx_ = *e* ^−18.375 ± 0.334^ *O*_cx_^1.022 ± 0.017^; (**c**) *M*_cb_ = *e* ^−19.529 ± 0.569^ *O*_cb_^1.074 ± 0.030^; (**d**) *M*_rob_ = *e* ^−19.186 ± 0.435^ *O*_rob_^1.058 ± 0.022^. The dotted lines indicate the 95% prediction interval. All values for the minke whale fit the predictions for a generic mammalian species.

We estimated that within the entire brain of the northern minke whale studied, there were 173.4 × 10^9^ non‐neuronal cells, of which 133.5 × 10^9^ non‐neuronal cells were found in the cerebral cortex, 22.3 × 10^9^ non‐neuronal cells in the CB, and 17.3 × 10^9^ non‐neuronal cells in the ROB (Table 1). The relationship between brain mass and the total number of non‐neuronal cells in the brain across all mammals studied to date with the isotropic fractionator method (Azevedo et al. 2009; Gabi et al. 2010; Herculano and Kaas 2011; Kazu et al. 2014; Herculano‐Houzel et al. 2007; 2011, Herculano‐Houzel, Manger, et al. 2014; Herculano‐Houzel, Catania, et al. 2015; Dos Santos et al. 2017; Jardim‐Messeder et al. 2017) is a power function with an exponent of 1.053 ± 0.016 (*r* ^2^ = 0.990, *p* < 0.0001; Figure 11a), very close to linearity. The data for the northern minke whale study falls well within the 95% prediction interval derived from this regression (Figure 11a), showing that the minke whale brain has the number of non‐neuronal cells predicted for a generic mammal of similar brain mass. Similar results are found when breaking down the brain into its main divisions: The cerebral cortex (Figure 11b), CB (Figure 11c), and ROB (Figure 11d). Thus, the whole brain and all major brain regions analyzed (cerebral cortex, CB, and ROB) of the northern minke whale follow the same relationship between structure mass and numbers of non‐neuronal cells that applies to all mammalian species examined to date (Herculano‐Houzel and Dos Santos 2018).

**FIGURE 12:**
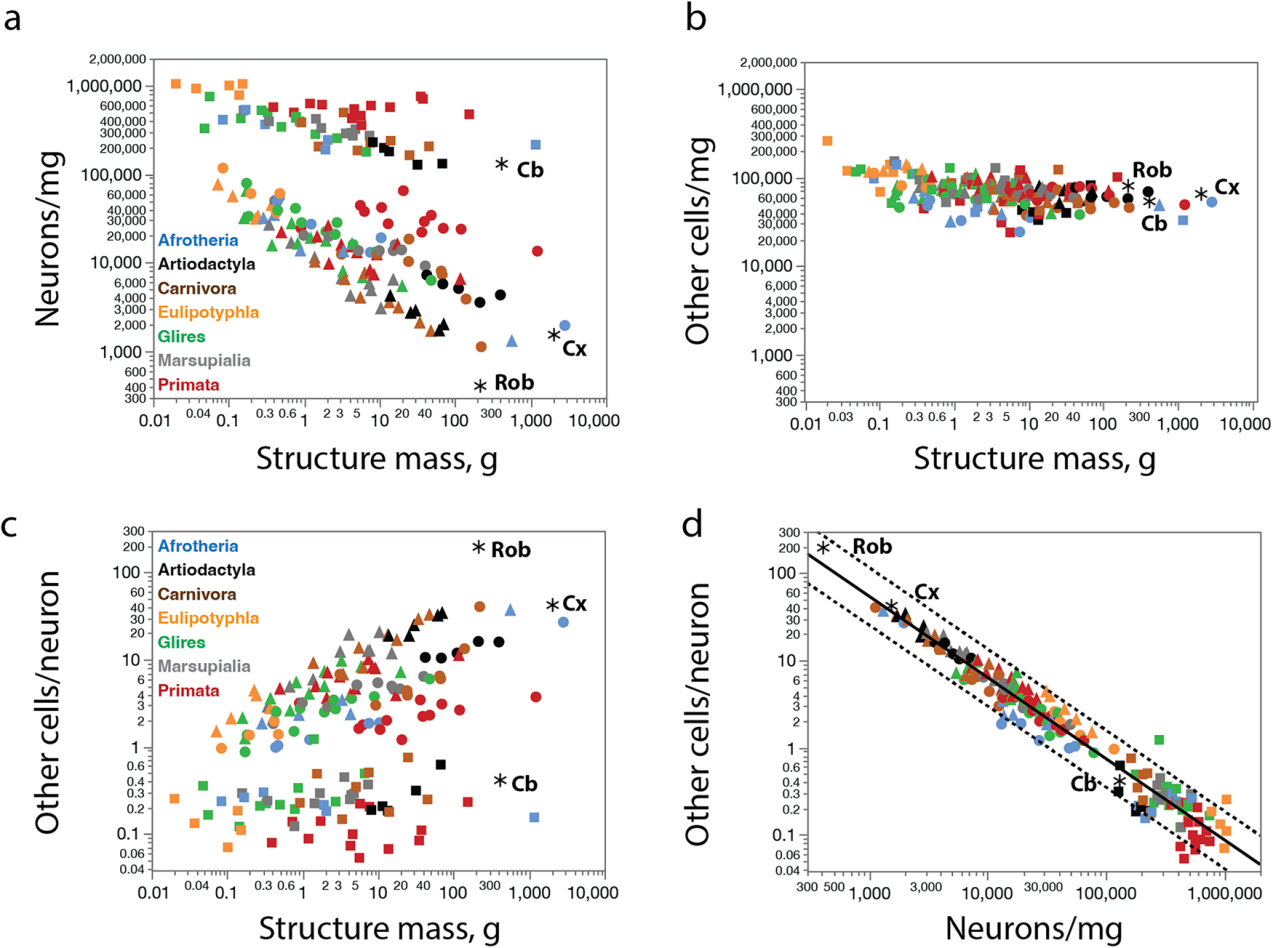
**The minke whale brain has the density of non‐neuronal (other) cells expected for a generic mammalian brain in each of its main divisions, and the ratio of non‐neuronal (other) cells/neuron expected for the neuronal density in each structure**. Species are shown as in the previous graphs, with the main brain divisions identified by the symbols: Cerebral cortex as circles, cerebellum as squares, rest of brain as triangles. Graphs show the large but systematic variation in neuronal density across non‐primate species and brain structures (**a**), in contrast to the small variation in other cell density in the same brain structures and species (**b**). Function plotted in (**d**), which includes all mammalian species in the dataset, minus the minke whale, is O/N = *e* ^10.434 ± 0.176^ *DN* ^−0.932 ± 0.016^. The dotted lines indicate the 95% prediction interval. All values for the minke whale fit the predictions for a generic mammalian species.

The mathematical consequence of the similar scaling relationships between numbers of non‐neuronal (other) cells and structure mass shown in Figure 11 for the major divisions of the brain is that, in contrast to the large (though orderly) variation in neuronal density (Figure 12a), the various brain structures and species share a similar, narrow range of non‐neuronal cell densities (Figure 12b). Importantly, the non‐neuronal cell densities we found in the minke whale cerebral cortex, CB, and ROB fall in the same narrow range as in other mammalian species (Figure 12b). As a consequence of the large variation in neuronal densities, but not in the densities of other cells, the ratio between the two cell types, which serves as an approximation of the glia/neuron ratio, is enormously variable, though not as a universal function of structure mass (Figure 12c) but rather of neuronal density in the structure (Figure 12d). We find that minke whale brain structures have some of the largest O/N ratios (Table 1), but with values that still match the predicted for the neuronal density in each structure (Figure 12d).

### 3.7 Distribution of Neuronal and Non‐Neuronal Cellular Densities Within the Northern Minke Whale Cerebral Cortex

**FIGURE 13:**
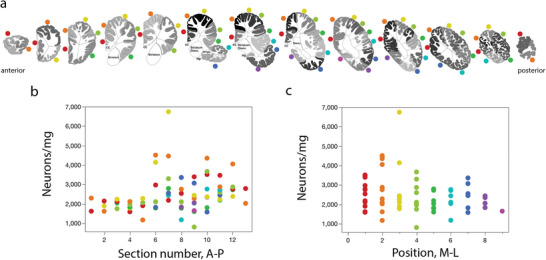
Distribution of neuronal and non‐neuronal cell densities in the northern minke whale throughout the cerebral cortex. Dorsal to ventral cortical blocks are color coded. (**a**) Neuronal density (neurons/mg) varies along the anterior‐posterior axis of the cortical grey matter (0.004 ± 0.001, *r* ^2^ = 0.096, *p* = 0.007; Spearman *ρ* = 0.485, *p* = 0.002), with a slightly higher density towards the posterior portion of the cortex. (**b**) Neuronal density also varies along the dorsal‐ventral axis of the cerebral cortex, with a higher density dorsally (−0.034 ± 0.015, *r* ^2^ = 0.064, *p* = 0.03; Spearman *ρ* = −0.251, *p* = 0.031). (**c**) The density of non‐neuronal cells does not vary along the anterior‐posterior axis throughout the grey and white matter of the cerebral cortex (*p* = 0.580). (**d**) The ratio of the number of non‐neuronal cells/neurons, decreases with increasing neuronal density (−0.784 ± 0.062, *r* ^2^ = 0.687, *p* = 0.001; Spearman *ρ* = −0.806, *p* = 0.001).

Dissection of the northern minke whale cerebral cortex in separate blocks of tissue allowed us to process the white and grey matter separately from each other, along the antero‐posterior axis, and across blocks within each coronal plane, to examine whether there were any gradients of neuronal density along the main axes of the cortex. Local neuronal densities are shown in Figure 1, and the full series of coronal sections of the cerebral cortex is rearranged in Figure 13a, color‐coded for the medial‐lateral position of each block. We found an average neuronal density in the cerebral cortical gray matter of the northern minke whale of 2367 ± 87 neurons/mg, with a local maximum of 4510 neurons/mg and a minimum of 1177 neurons/mg. The neuronal density exhibits a gradient along the anterior–posterior axis, with significantly higher densities of neurons towards the posterior pole of the cortex (Spearman *ρ* = 0.378, *p* = 0.0011; Figure 13b). Intriguingly, there seems to be an abrupt change in neuronal density at the coronal level where the temporal lobe becomes visible, in the sixth section of the series (Figure 13a,b). While the medial cortical areas at this level have strikingly higher neuronal densities than more lateral areas (Figure 13a), there was no significant gradient of neuronal density along the medial–lateral expansion of the cerebral cortex (Spearman *ρ* = −0.214, *p* = 0.0710; Figure 13c).

## 4 Discussion

Using the isotropic fractionator, the same method that we have been employing to build a database of the cellular composition of over 200 vertebrate brains, here we confirm our prediction that the minke whale brain conforms to the scaling rules that apply to the cellular composition of artiodactyl brains (Kazu et al. 2014). We find that the minke whale cerebral cortex, despite having about twice the mass of the human cerebral cortex, has only 3.2 billion neurons, as expected for a generic artiodactyl cortex of its mass, compared to the average of 16 billion neurons that we found in the human cerebral cortex, also the number expected for a generic primate of our cortical mass (Azevedo et al. 2009). The finding that the large minke whale cerebral cortex only contains the 3.2 billion neurons expected for an artiodactyl, and not the 12.8 billion neurons estimated previously with stereology (Eriksen and Pakkenberg 2007), provides crucial new information to the debate on cetacean cognitive capabilities, which we discuss below. We also address below the probable cause of the overestimate obtained with the stereological approach.

Here we show that in many quantitative aspects—neuronal densities in the cerebral cortex and CB, numbers of neurons in these structures compared to their mass, non‐neuronal cell densities and non‐neuronal/neuronal cell ratios overall—the minke whale brain is a scaled‐up, generic artiodactyl brain. What distinguishes the minke whale in terms of the quantitative cellular composition of its brain is: (1) The approximately 4 times larger ratio of cerebellar to cerebral cortical neurons compared to all other mammalian species examined to date, with the exceptions of the African elephant and the brown bear (Herculano‐Houzel, Avelino‐de‐Souza, et al. 2014; Jardim‐Messeder et al. 2017); and (2) the 60% fewer neurons than predicted in the ROB for their ROB mass, and for the numbers of neurons in the cerebral cortex and CB, with a neuronal density in the ROB that is extraordinarily low even for the large size of this structure, although still within the prediction interval for a generic non‐primate mammal of its body size. The demonstration that the artiodactyl scaling rules, and indeed in many aspects the non‐primate mammalian scaling rules, apply to the minke whale brain warrants using these scaling rules to make predictions about the cellular composition of the brain of other cetaceans, which we pursue below.

### 4.1 Consistency of Current Isotropic Fractionator Results With Previous Findings

The exceptionally low neuronal densities in the ROB of the northern minke whale might suggest a failure of our method to assess the numbers of cells in this part of the brain correctly, and a similar argument might be made to question our lower estimate of 3.2 billion neurons in the minke whale cortex compared to the stereological estimates for this and other cetaceans in the literature. We can confidently reject this possibility for several reasons.

First, although our estimate of a grand average of 2367 neurons/mg in the grey matter of the cerebral cortex of the minke whale is lower than previous estimates for cetacean species, it matches the density expected for a cetartiodactyl cortex of its mass, and indeed the density expected for any non‐primate mammalian species. In contrast, the previously reported neuronal densities of upwards of 10,000 and up to 30,900 neurons/mg in the grey matter of the cerebral cortex of cetaceans (in the case of the pilot whale; Mortensen et al. 2014) are more than an entire order of magnitude higher than expected, as can be seen in Figure 5. Using the cortical grey matter mass of 1136.5 g reported here, a minke whale with the 12.8 billion cortical neurons estimated by Eriksen and Pakkenberg (2007) would have an overall average density of 11263 neurons/mg. Such neuronal densities would indeed make whales massive outliers in brain evolution. Harbor porpoise and bottlenose dolphin presumed primary sensory areas and primary motor cortex have 12,000–40,000 neurons/mg in layers III and V, which are the densest layers of the cetacean cortical grey matter (Kern et al. 2010). While similar neuronal densities were reported in layers III and V of three other species, with even higher densities in Layer II (Ridgway et al. 2019), Layer I, which is relatively thick in the cetacean cortex, has lower densities similar to those reported here (Ridgway et al. 2019). Estimates of neuronal density restricted to layers II, III, and V are therefore overestimates of what applies to the grey matter as a whole. In that regard, Ridgway et al. (2019) report “area means” of 11,000–20,000 neurons/mg, but unfortunately, those “area means” are not mathematically correct, for they are a simple mean of means across the cortical layers counted, and do not account for the large thickness of the low‐density Layer I. Indeed, the earliest estimates, using direct counting of the entire cerebral cortical wall, reported lower densities of 6500–7000 neurons/mg in the fin whale (Tower 1954), which would still fall within the 95% prediction interval reported here (Figure 5).

Thus, our estimates for the minke whale cerebral cortex and CB meet the internal consistency of the numbers we obtained with the same method, which supports the existence of scaling relationships that apply to the ensemble of mammalian species analyzed as expected for evolutionary rules that apply to the vast majority of species. In regard to the cerebral cortex, there are only two exceptions reported to date: The raccoon, with twice the number of neurons expected in all subdivisions of its brain, and the brown bear, with only about one‐fourth the number of neurons expected for its cortical mass (Jardim‐Messeder et al. 2017). We have speculated that the latter is due to neuronal loss related to the energetic insufficiency that drives brown bears into hibernation. At this point, we do not believe that this is the cause of the low numbers of ROB neurons in the minke whale, since this species, unlike the brown bear, does not show evidence of energetic insufficiency. Future studies on other whale species will show whether this is particular to the minke whale or a general property of whale brains.

Second, our finding of non‐neuronal cell densities in the minke whale brain, well within the predicted range in the cerebral cortex, CB, and ROB alike (Herculano‐Houzel, Catania, et al. 2015; Jardim‐Messeder et al. 2017), speaks to the consistency and accuracy of our method. Of note, non‐neuronal cell densities are also similar in the cerebral cortex of the brown bear and raccoon, and in the CB of the elephant and the brown bear, with the few cases of neuronal densities outside the predicted interval in our dataset. Moreover, given our estimate that endothelial cells are one‐third of all non‐neuronal cells in any brain tissue (Ventura‐Antunes et al. 2022), our estimate of 133.5 billion non‐neuronal cells in the minke whale cortex, which also includes endothelial cells, is an excellent match to the previous stereological estimate in the literature of 98.2 billion glial cells in the neocortex of the same species (Eriksen and Pakkenberg 2007). This finding alone suggests that the divergence in neuronal numbers between ours and the stereological estimate for the minke whale cortex, as well as the low neuronal density we find in the ROB, is not due to counting error or failure of the isotropic fractionator to capture cell counts accurately.

Third, as a consequence of the first two points, the ratios between non‐neuronal (other) and neuronal cells that we report here match the O/N ratios predicted for the neuronal densities observed in the different brain structures, including the cerebral cortex. Our finding of an O/N ratio of 17.6 in the grey matter of the minke whale cerebral cortex is even higher than the values previously reported in the fin whale (4.5:1–5.9:1, Hawkins and Olszewski 1957) and bottlenose dolphin (2:1–3:1, Garey and Leuba 1986), but is consistent with the low neuronal density we report. We have shown previously that the glia/neuron ratio is not, as formerly believed, an inverse function of body or brain mass, but rather a very tight function of the scaling of neuronal density in the face of a relatively constant density of other cells (Mota and Herculano‐Houzel 2014). Thus, the number of neurons and glial cells reported for the common minke whale using stereology (Eriksen and Pakkenberg 2007), amounting to a G/N ratio of 7.7, still agrees with what we argue to be an overestimate of neuronal density of about 11,000 neurons/mg in the minke whale cortical grey matter, given our similar estimates of non‐neuronal cell densities. This leads to the crucial point for the comparison of our data to the previous estimate: Why do our estimates for non‐neuronal cells match the stereological estimate for the minke whale of Eriksen and Pakkenberg (2007), but not their estimated numbers of neurons?

### 4.2 Why Are Stereological Estimates of Numbers of Cortical Neurons in Cetaceans so High?

The cerebral cortex is thought to be the brain region that is involved in higher cognitive functions, with the number of neurons within the cerebral cortex potentially forming one of the principal foundations for the level of complexity of neuronal information processing that can be achieved (Herculano‐Houzel 2016). In this context, it is important to compare the current estimates of cortical neuronal numbers in the northern minke whale brain with those published previously (Eriksen and Pakkenberg 2007). In the current study, using the isotropic fractionator technique, we estimate that there are approximately 3.2 × 10^9^ cortical neurons in the northern minke whale. The study of Eriksen and Pakkenberg (2007), using stereological techniques, provided an estimate of 12.8 × 10^9^ cortical neurons, around 4 times more cortical neurons than the current study. We suspect that the discrepancy in these estimates using the isotropic fractionator compared to stereological techniques is due to undersampling with stereological techniques (Herculano‐Houzel, Kaas, et al. 2015).

Eriksen and Pakkenberg (2007) report that only 12–13 sections out of over 3000 sections of the northern minke whale cerebral cortex were sampled, counting only up to 215 neurons per brain. It has unfortunately been a consistent problem with reports on cetacean brains that the details necessary to evaluate the adequacy of the stereological parameters used are not provided, such as the size of the counting frame, the total number of frames and cells counted, or the average number of cells counted per frame. Assuming ca. 20 counting frames placed on each section, as shown in Figure 2 of that report, gives a total of 240–260 counting frames for 215 neurons at most, with an average of less than one neuron per frame. In other words, the counting frames that they used were so small that they may or may not include a neuron (while they will still include about one order of magnitude as many non‐neuronal cells, given their much higher density, providing a proper estimate for these cells). Similar undersampling was used in the other reports on cetacean neuronal densities reviewed here. Making estimates from large brains with highly heterogeneous neuronal densities, as we show here, by sampling from a few and minute sites, makes estimates of total numbers of neurons sensitive to the size of the brain structure counted relative to the minute size of the counting frame. This sensitivity to the volume of the structure analyzed defeats the purpose of unbiased stereological estimates (Herculano‐Houzel, Kaas, et al. 2015). Moreover, the sparse sampling from the entire cortex does not account for regional variations in neuronal density as reported herein (Figure 13), and completely ignores the effect of the relative size of the cortical regions bearing such different densities on the estimate of total numbers of cortical neurons.

In contrast, the isotropic fractionator method quantifies large (or small) brain structures by collecting samples from the structure of interest only once it has been turned homogeneous. This eliminates undersampling errors as well as errors related to variations in neuronal density and, most importantly, any possibility that the estimate obtained is biased by the original volume of the structure (Herculano‐Houzel and Lent 2005). Further, in using the isotropic fractionator, the problem of dealing with a Poisson distribution is eliminated by observing good cell counting practice with a hemocytometer chamber that requires a minimum of 50–60 objects per field counted. Most importantly, side‐by‐side comparison with unbiased stereological sampling that followed the guidelines to avoid undersampling has validated the isotropic fractionator counting technique (Miller et al. 2014; Bahney and von Bartheld 2014), further strengthening the results provided herein.

### 4.3 The Problem of Sample Limitation to a Single Specimen

As detailed in the Methods, the findings we describe here refer to a single specimen of minke whale, which is evidently less than ideal, for it does not allow confirmation across specimens, much less the investigation of individual variation. This is a limitation that cannot be easily overcome for the obvious reason that having access to freshly deceased whale individuals, much less with the conditions to collect their brains, is a matter of opportunity and luck. Our findings on the brown bear and the African elephant, and a number of other mammalian species, have similarly been based on single individuals out of necessity. We acknowledge, therefore, the possibility that, by pure chance, the individuals analyzed here and in previous studies are not representative of their species.

On the other hand, the point of the present analyses, as in our related studies on brain scaling, is to investigate the patterns that apply to the cellular composition of brains that vary across several orders of magnitude in size and numbers of cells. As indicated above, we find that the specimen we could analyze here conformed in most ways to the cellular scaling rules that apply to its order, Cetartiodactyla, which rules out gross widespread changes that could be due, for example, to disease or congenital abnormalities. In addition, where we find that the minke whale brain analyzed here deviates from the applicable scaling rules, that deviation is several‐fold, well beyond the range of ca. 50% variation around the mean that we find to apply across individuals of a non‐isogenic strain of mice (Herculano‐Houzel, Messeder, et al. 2015). We thus find it likely that the present findings are indeed representative of the species investigated within the context of scaling relationships spanning several orders of magnitude.

### 4.4 A Role of Non‐Neuronal Cell Numbers in Thermogenesis?

The current study finds the highest non‐neuronal/neuronal cell ratio in the cerebral cortex of the northern minke whale, compared to all other species, including Cetartiodactyla, where numbers have been estimated with the isotropic fractionator method. Importantly, this high ratio is just what is expected for the low neuronal density of the northern minke whale cerebral cortex (Mota and Herculano‐Houzel 2014; Herculano‐Houzel and Dos Santos 2018), which indicates that the large non‐neuronal/neuronal cell ratio in this cortex is not the product of selection for or against itself. To the contrary, this universal scaling relationship between structural mass and the numbers of non‐neuronal cells appears to be a fundamental constraint of the mammalian brain, one that the current analysis suggests has not been significantly modified during the transition to an obligatory aquatic life history, other neurological changes that have occurred in cetacean brains notwithstanding (Manger 2006; Patzke et al. 2015).

Despite not being an outlier in cortical scaling, the high non‐neuronal/neuronal ratio in cetacean brains might still play an important role in cetacean brain physiology and evolution. The validity of the increased ratio of non‐neuronal/neuronal cell ratios with decreasing neuronal densities throughout mammalian brain evolution means that larger cetacean brains evolved with higher and higher glia:neuron ratios. This finding is of interest in light of the expression of uncoupling proteins 4 and 5 (UCPs), thought to be involved in non‐shivering neurothermogenesis, in ∼56% of glial cells in the subcortical white matter and ∼36% of glial cells in the cortical grey matter of the minke whale (Manger et al. 2021). Interestingly, UCPs, while present in the brain of terrestrial cetartiodactyls (as shown with Western immunoblotting), are not specifically localized in the glial cells in those brains (Manger et al. 2021). Thus, following the logic of the adaptive neurothermogenetic proposal of large brain evolution in cetaceans (Manger 2006; Manger et al. 2021), it could be speculated that a substantial proportion of the glia in the ancestral Neocete brain became specialized for neurothermogenesis (approximately 32 mya, Manger 2006), with the cetartiodactyl trait of higher glia:neuron ratios that come with their low neuronal densities associated with larger numbers of neurons providing the morphophysiological basis for this potential adaptive value of high glia/neuron ratios that accompany larger numbers of neurons in cetacean brains. In other words, thermoregulation of the brain in cold aquatic environments may have been a significant factor driving the evolution of larger brains in cetaceans.

### 4.5 Estimating the Cellular Composition of Other Cetacean Brains

As addressed above, the interpretive capacity of the current study is limited by the analysis of a single specimen, given the opportunistic nature of the collection, which, of course, allows no insight into the range of variation of cell numbers across individuals of this species. However, the intraspecific variation that can be expected to be on the order of 20%–50% (Herculano‐Houzel, Messeder, et al. 2015), which pales in comparison to the scaling across species, and to the discrepancy with previous stereological estimates. Keeping this caveat in mind, the consistency of the findings reported with the findings for other cetartiodactyl species is strongly supportive of the present estimates being reliable and representative for the species as a whole for the purposes of studies of evolutionary brain scaling across several orders of magnitude in brain and body size. Although our conclusions must be tempered with this limitation in mind, given the lack of availability of cetacean brain material prepared appropriately for this, and other, modern neuroanatomical methods, we can make the assumption, as a working hypothesis for the purposes of discussion of the results and their implications, that the data presented herein are generally representative of the Mysticeti parvorder of the Cetacea. We can go further and postulate that these results are also representative of the infraorder Cetacea as a whole, including Odontoceti, given the broad similarities in brain structure across the Cetacea (Manger 2006; Patzke et al. 2015), although further analyses of other cetacean species are required to confirm this assumption and generalize the results to the entire infraorder.

Given this caveat and the assumptions stated, we can predict how many neurons compose the cerebral cortex of other cetacean species of known brain mass, using the equation *N*_cx_ = *e* ^17.052^ *M*_brain_^0.600^ that applies to cetartiodactyls (Herculano‐Houzel 2019). These predictions are listed in Table 2. Importantly, the extension to cetaceans of the scaling rules that apply to artiodactyls means that even large‐brained species such as the blue whale, the fin whale, and the killer whale, with brains weighing between 6 and 7 kg, still have an estimated number of cortical neurons around 5 billion, on par with the African elephant and below great apes.

**TABLE 2:** **Actual and predicted numbers of cortical neurons calculated from brain mass using the isotropic fractionator**. Species are listed in order of decreasing brain mass. Predictions were made given the equations that apply to cetartiodactyls (*N*_cx_ = *e* ^17.052^ *M*_brain_^0.600^), to afrotherians (*N*_cx_ = *e* ^16.965^ *M*_brain_^0.651^), and to primates (*N*_cx_ = *e* ^17.125^ *M*_brain_^0.933^) reported in Herculano‐Houzel (2019). *N*_cx_, total number of neurons in the cerebral cortex; *M*_brain_, brain mass in grams. References for actual *N*_cx_ obtained with the isotropic fractionator are (1) Herculano‐Houzel, Manger, et al. 2014; (2) Azevedo et al. (2009); (3) Kazu et al. (2014); (4) Miller et al. (2014); (5) Gabi et al. (2010); and (6) Herculano‐Houzel et al. (2007).

Species	Common name	Brain mass (g)	Predicted *N*_cx_	Actual *N*_cx_
*Balaenoptera physalus*	Fin whale	7,100	5.2 billion	
*Balaenoptera musculus*	Blue whale	6,800	5.1 billion	
*Orcinus orca*	Killer whale	6,052	4.7 billion	
*Loxodonta africana*	African elephant	4,420	5.5 billion	5.6 billion (1)
*Globicephala macrorhyncha*	Short‐finned pilot whale	2,818	3.0 billion	
*Balaenoptera acutorostrata*	Minke whale	2,684	2.9 billion	3.2 billion
*Delphinapterus leucas*	Beluga	2,354	2.7 billion	
*Phocoena phocoena*	Harbor porpoise	1,735	2.2 billion	
*Homo sapiens*	Human	1,460	24.5 billion	16.3 billion (2)
*Hipopotamus amphibius*	Hippopotamus	720	1.3 billion	
*Giraffa camelopardalis*	Giraffe	700	1.3 billion	1.7 billion (3)
*Gorilla gorilla*	Gorilla	574	10.3 billion	
*Pan troglodytes*	Chimpanzee	449	8.2 billion	6–7 billion (4)
*Pongo pygmaeus*	Orangutan	422	7.7 billion	
*Papio anubis*	Baboon	152	3.0 billion	2.9 billion (5)
*Macaca mulatta*	Rhesus monkey	93	1.9 billion	1.7 billion (6)

### 4.6 The Minke Whale Brain Has an Enlarged Proportion of Cerebellar to Cerebral Cortical Neurons

Here we find that the minke whale brain analyzed had a high ratio of 17 cerebellar neurons to every cerebral cortical neuron, about 4 times as high as the mammalian average of 4.1. This result was unsuspected, given the relative mass of the CB being 15.5% of brain mass, which, while significantly larger than the 10% and 14% of total brain volume or mass that the CB typically represents in mammals (Clark et al. 2001; Herculano‐Houzel 2010), is still fairly small. Such a high ratio of neurons between cerebellar and cerebral cortices is so far only surpassed by the African elephant in our dataset (Herculano‐Houzel, Manger, et al. 2014). Assuming that corticocerebellar circuits are involved in cognition, we have proposed that any “extra” cerebellar neurons beyond those in proportionality to the number of cerebral cortical neurons are related to sensorimotor functions, not cognition (Herculano‐Houzel, Manger, et al. 2014). We had previously speculated that the strikingly high ratio of 46 cerebellar neurons for every cerebral cortical neuron in the African elephant was associated with the highly mobile, muscular trunk (Herculano‐Houzel, Manger, et al. 2014), although a role in specialized infrasonic communication is also possible. The exceptionally high ratio of cerebellar to cortical neurons in the minke whale might be associated with a variety of sounds and clicks used in navigation, even though baleen whales are generally thought to be incapable of echolocation. We urge caution in making that conclusion, however, given our finding that the relatively large CB of echolocating microbats still contains the expected ratio of cerebellar to cerebrocortical neurons (Herculano‐Houzel et al. 2020).

The minke whale and the African elephant also share a disproportionately large number of cerebellar neurons for the number of neurons found in their ROB. Although partly explained by the increased absolute number of cerebellar neurons compared to the expected for the cerebral cortex, there is a second contributing factor to this ratio in the minke whale, which is the unexpectedly small number of neurons in the ROB for the size of the structure as well as for the size of the body, along with exceptionally low neuronal density, both well below the 95% prediction interval. Thus, we find fewer neurons than expected forming the non‐cortical neuronal circuits crucial for processing information related to sensory perceptions, planning and execution of actions, such as the ascending sensory and descending motor pathways, the thalamocortical loop, the corticostriatal pathways, and the cortico‐cerebellar circuit—although at present we cannot differentiate among these circuits. Interestingly, even if comprised of fewer than expected neurons for a non‐primate mammal, these circuits obviously remain functional enough for behavior, with as many ROB neurons as found in other (smaller) cetartiodactyls species, which is in line with our previous observation that larger bodies do not necessarily require more ROB neurons to operate them (Watson et al. 2013; Ngwenya et al. 2016). We suggest that the extraordinarily low number of neurons in the ROB is related to the absence of limbs in modern cetaceans. Examination of the ROB of other cetacean species will determine whether this is a cetacean feature or a particularity of the northern minke whale.

### 4.7 Cognitive Implications

**FIGURE 14:**
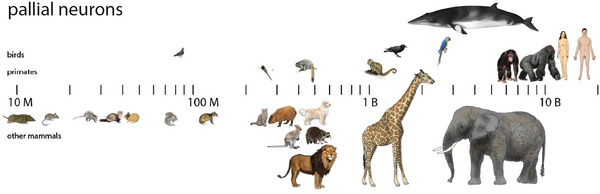
**Ranking of mammal and bird species by total numbers of neurons in the pallium/cerebral cortex places the minke whale between monkeys and great apes**. The center of each illustration is aligned to the number of pallial neurons in that species estimated using the isotropic fractionator. All illustrations by Lorena Kaz, except for the minke whale, from Shutterstock.

To date, the strongest neuroanatomical correlate of cognitive capacities across vertebrate species is the absolute number of neurons in the pallium (Herculano‐Houzel 2017), and in the associative regions of the pallium in particular (Ströckens et al. 2022). The number of cortical neurons in the Northern minke whale is approximately one‐third of the 8–9 billion neurons estimated to compose the cerebral cortex of the orangutan and gorilla (Herculano‐Houzel and Kaas 2011). All else being equal in terms of connectivity, and noting the caveat that the fraction of cortical neurons that are associative in function may differ between cetaceans and primates, our data place the expected cognitive capacity of the northern minke whale, as well as other large‐brained cetaceans (Table 2), between that of monkeys and great apes (Figure 14). Thus, while cetaceans certainly stand out amongst animals in their large absolute numbers of cortical neurons, the estimates provided in Table 2 go against claims that their intellectual capacity is second only to humans (Reiss et al. 1997; Marino 1996, 1998, 2004; Marino et al. 2008, 2007; Connor 2007; Grimm 2010; Herman 2012). Importantly, these popular claims lack solid behavioral evidence to equate cetaceans and great apes in terms of cognitive capabilities (Manger 2013).

A simple association between absolute total numbers of cerebral cortical neurons and the cognitive capacities of different mammalian species relies on the assumption that neurons are equally capable in their signal processing capabilities across species. Previous studies of individual cortical neurons in the northern minke whale showed a smaller total dendritic length, extent of dendritic branching, and number of dendritic spines than observed in the much smaller‐brained but phylogenetically affiliated giraffe (Jacobs et al. 2015). On a per‐neuron basis, a comparatively lower number of dendritic spines and lower total dendritic length should reduce the potential for northern minke whale cortical neurons to receive information, limiting the potential for sampling diversity of incoming neuronal information from other cortical and non‐cortical structures. Similarly, the comparative decrease in the extent of dendritic branching should lower the amount of local information processing occurring in the dendritic branches, thereby homogenizing the summation and processing of the dendritic potential, leading to less complexly processed dendritic potentials reaching the soma. Moreover, as the cetacean brain evolved from an ancestral artiodactyl brain, with its inherent “artiodactyl‐like” organization (Manger 2005), that is quite different from the “primate‐like” brain organization, direct comparisons of generalized cognitive capacities of cetaceans and primates would require crucial assumptions being made that to date have no clear scientific support.

An interesting further complication is the finding, mentioned above, that all cortical neurons in the northern minke whale contain UCP1, as opposed to around 35% in artiodactyls (Manger et al. 2021). This finding implies that the cetacean cortical neurons, which contain a high density of mitochondria (Morgane et al. 1990), are likely to expend a substantial portion of their energy and time in the production of heat rather than on the typical functions associated with neurons, which might compromise the signal processing capabilities of cetacean cortical neurons. On the other hand, while we now know that large neurons receive more energy per neuron than smaller ones in the adult brain (Ventura‐Antunes and Herculano‐Houzel, 2022; Ventura‐Antunes et al. 2022), it remains to be determined how energy availability and budgets per neuron compare across species. Thus, while the number of neurons in the northern minke whale cerebral cortex is what would be expected for a cetartiodactyl with a ∼2.6 kg brain, they may or may not have the systems level processing power and dendritic hardware to facilitate neuronal information flow in such a way as to produce cognitive outcomes that would rival that achieved by similar numbers of cortical neurons in the brains of primates.

## Author Contributions

Paul R. Manger and Karl Æ. Karlsson collected tissue. Nina Patzke, Suzana Herculano‐Houzel, and Paul R. Manger designed research. Kamilla Avelino‐de‐Souza, Nina Patzke and Suzana Herculano‐Houzel processed the tissue, analyzed data, and wrote the first draft of the manuscript. Suzana Herculano‐Houzel and Paul R. Manger edited the manuscript.

## Conflicts of Interest

Suzana Herculano‐Houzel, one of the corresponding authors of this article, is the Editor‐in‐Chief of this journal. She was excluded from the peer‐review process and all editorial decisions related to the acceptance and publication of this article. Peer‐review was handled independently by JCN/CNE Journal editorial office and Dr. Anna Roe as Editor to minimize bias. The remaining authors declare no conflicts of interest.

## Peer Review

The peer review history for this article is available at https://publons.com/publon/10.1002/cne.70089

## Data Availability

The data that support the findings of this study are available from the corresponding author upon reasonable request.
